# A TMT-based shotgun proteomics uncovers overexpression of thrombospondin 1 as a contributor in pyrrolizidine alkaloid-induced hepatic sinusoidal obstruction syndrome

**DOI:** 10.1007/s00204-022-03281-7

**Published:** 2022-03-31

**Authors:** Weiqian Wang, Yan Chen, Yue Yin, Xunjiang Wang, Xuanling Ye, Kaiyuan Jiang, Yi Zhang, Jiwei Zhang, Wei Zhang, Yuzheng Zhuge, Li Chen, Chao Peng, Aizhen Xiong, Li Yang, Zhengtao Wang

**Affiliations:** 1 The MOE Key Laboratory for Standardization of Chinese Medicines and the SATCM Key Laboratory for New Resources and Quality Evaluation of Chinese Medicines, Institute of Chinese Materia Medica, Shanghai University of Traditional Chinese Medicine, Shanghai, 201210 China; 2 Shanghai R and D Center for Standardization of Traditional Chinese Medicines, Shanghai, 201210 China; 3 Institute of Interdisciplinary Integrative Medicine Research, Shanghai University of Traditional Chinese Medicine, Shanghai, 201210 China; 4 National Facility for Protein Science in Shanghai, Shanghai Advanced Research Institute, Chinese Academy of Science, Shanghai, 201210 China; 5 Department of Gastroenterology, The Drum Tower Hospital of Nanjing, affiliated to Nanjing University Medical School, Nanjing, 210008 Jiangsu China; 6 Department of Gastroenterology, School of Medicine, Ruijin Hospital, Shanghai JiaoTong University, Shanghai, 201801 China

**Keywords:** Pyrrolizidine alkaloid, Hepatic sinusoidal obstruction disease, Hepatic sinusoidal endothelial cells, TMT-based shotgun proteomics, Thrombospondin 1, Pyrrole-protein adduct

## Abstract

Hepatic sinusoidal obstruction disease (HSOS) is a rare but life-threatening vascular liver disease. However, its underlying mechanism and molecular changes in HSOS are largely unknown, thus greatly hindering the development of its effective treatment. Hepatic sinusoidal endothelial cells (HSECs) are the primary and essential target for HSOS. A tandem mass tag-based shotgun proteomics study was performed using primary cultured HSECs from mice with HSOS induced by senecionine, a representative toxic pyrrolizidine alkaloid (PA). Dynamic changes in proteome were found at the initial period of damage and the essential role of thrombospondin 1 (TSP1) was highlighted in PA-induced HSOS. TSP1 over-expression was further confirmed in human HSECs and liver samples from patients with PA-induced HSOS. LSKL peptide, a known TSP1 inhibitor, protected mice from senecionine-induced HSOS. In addition, TSP1 was found to be covalently modified by dehydropyrrolizidine alkaloids in human HSECs and mouse livers upon senecionine treatment, thus to form the pyrrole-protein adduct. These findings provide useful information on early changes in HSECs upon PA treatment and uncover TSP1 overexpression as a contributor in PA-induced HSOS.

**Graphical abstract:**

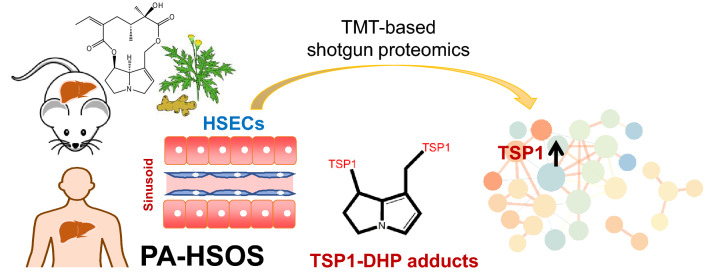

**Supplementary Information:**

The online version contains supplementary material available at 10.1007/s00204-022-03281-7.

## Introduction

Hepatic sinusoidal obstruction disease (HSOS), also known as hepatic veno-occlusive disease, is a rare but life-threatening vascular liver disease that is clinically characterized by the detachment of endothelial cells in small sinusoidal hepatic and interlobular veins and usually presents as portal hypertension followed by parenchymal dysfunction (Helmy 2006; Zhuge et al. 2019). HSOS is primarily caused by damage to hepatic sinusoidal endothelial cells (HSECs) and has been reported as early as 1920 in South Africa as a result of consuming of bread made from wheat contaminated by pyrrolizidine alkaloids (PAs) (Willmot and Robertson 1920). To date, over 8000 HSOS cases by PA exposure via food, health supplements, or herbal medicine such as *Gynura japonica* have been reported worldwide, including in South Africa, China, Germany, Spain, and the USA (IPCS 1988).

PAs are currently the most hepatotoxic natural compounds with global distribution. More than 660 PAs and their *N*-oxides have been identified in over 6000 flowering plants. Humans are often exposed to PAs by accidentally ingesting or consuming PA-containing medicinal herbs and teas, and PA-contaminated diets such as wheat and honey (IPCS 1988; EFSA 2017). PAs can be metabolized by cytochrome P450 enzymes (CYP450s) to form intermediate dehydropyrrolizidine alkaloids, which in turn can rapidly interact with cellular macromolecules such as proteins to generate pyrrole–protein adducts (PPAs) that initiate toxicity (Fu et al. 2004). Owing to the complexity of its pathogenesis, understanding the molecular mechanism of PA-induced HSOS remains a huge challenge and greatly hinders the development of effective clinical treatments.

Few proteomics studies have uncovered the potential molecular mechanism of PA-induced hepatotoxicity (Wang et al. 2012; Li et al. 2018). A proteomics research on isoline-induced acute liver injury employed traditional 2D gel electrophoresis combined with matrix-assisted laser desorption ionization (MALDI)-time of flight (TOF) mass spectrometry (MS) (Wang et al. 2012), which reveals several differentially expressed proteins involved in the process of oxidative stress or cellular energy metabolism. Li et al. (2018) suggested that seven proteins and three toxicity pathways of vascular endothelial cell death are associated with retrorsine-induced hepatotoxicity. All these works suggest the potential of utilizing proteomics in the mechanism study of PA-induced toxicity. However, these investigations were mainly based on traditional 2D gel electrophoresis combined with MALDI-TOF MS, which emphasize only proteins with high abundances. In addition, only the total protein lysates of the whole liver tissues, which consist of over 95% of parenchymal hepatocytes, were usually employed. All these disparities may result in the insufficient identification of certain proteins with low abundant but essential roles in PA-induced HSOS, especially those present in HSECs, where the initial and crucial pathogenic event of PA-induced HSOS occurs (DeLeve et al. 1999).

With the development of quantitative proteomics strategies, such as tandem mass tag (TMT) labeling combined with UPLC-MS/MS, shotgun proteomics has been increasingly used and showed great advantages over the traditional approach in increasing sample multiplexing for relative quantification, increased sample throughput, and reduced missing quantitative channels in the sample. The accuracy of the dynamic range of peptide mapping and protein level is more acceptable than other isobaric mass tags (Thompson et al. 2003; McAlister et al. 2012; Pappireddi et al. 2019). Therefore, a TMT-based shotgun proteomics study was performed in HSECs primary cultured from mice treated with senecionine, a well-known hepatotoxic PA compound. The protein signatures revealed by proteomics were also confirmed in human HSECs and patients with PA-induced HSOS. This study deciphers new molecular mechanisms underlying this disease and lays the scientific basis for the clinical treatment of PA-induced HSOS.

## Material and methods

### Chemicals and reagents

**Fig. 1:**
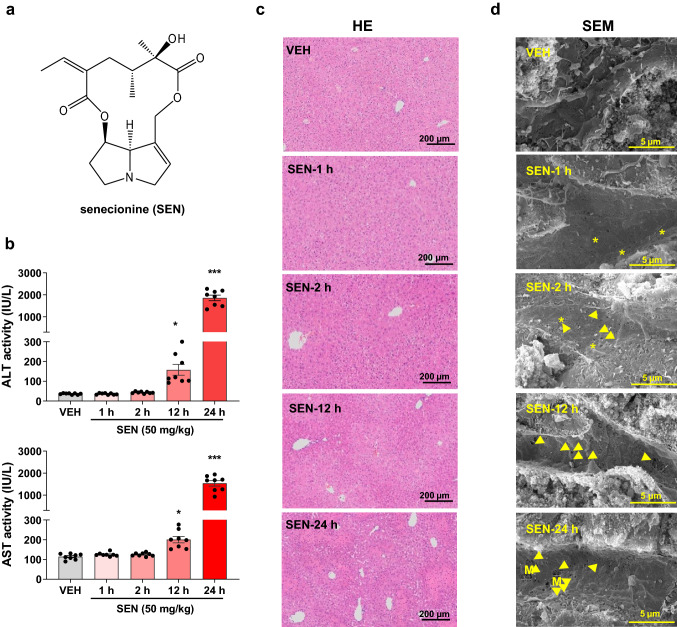
Senecionine induces severe liver injury in mice with initial damage in the hepatic sinusoid. Mice were orally treated with blank solvent (VEH) or senecionine (SEN, 50 mg/kg body weight) for 1, 2, 12, and 24 h. a Chemical structure of senecionine. b Serum ALT and AST activities (*n* = 8). Values are expressed as the mean ± SEM. *p < 0.05, **p < 0.01, ***p < 0.001 *vs.* VEH group. c Typical images of HE staining of liver tissues (*n* = 3). Scale bar: 200 µm. d Typical scanning electron microscopy images of liver tissues (*n* = 3). Scale bar: 5 µm. The yellow asterisk indicates the loss of fenestrae organized as sieve plates in sinusoidal. The yellow arrow indicates the formation of large gaps in the sinusoidal endothelium and exposure of the Disse space. M indicates the visible hepatocyte microvilli through the gap in HSECs (color figure online)

Senecionine with purity of over 98% was purchased from Chengdu Biopurify Phytochemicals, Ltd. (Sichuan, China) (Fig. 1a) and dissolved in acidified 0.9% sodium chloride (pH 6.5) to prepare a solution containing 5 mg of senecionine per mL for animal experiments. Senecionine was also dissolved in acidified phosphate buffer to obtain a solution of 500 mM and diluted with culture medium for cell culture treatment. LSKL TSP-1 inhibitory peptide was purchased from Glpbio (CA, USA) and dissolved in 0.9% sodium chloride to prepare a solution containing 3 mg of LSKL per mL for animal experiments.

Primary antibodies, including thrombospondin 1 (TSP1, #ab85762), matrix metallopeptidase 9 (MMP9, #ab228402), transforming growth factor-beta 1 (TGFB1, #ab179695), and CD47 (#ab175388), were bought from Abcam (Cambridge, MA, USA). Primary antibodies for TP53 (#2524) and β–Actin (#4970) were acquired from Cell Signaling Technology (Danvers, MA, USA). Secondary antibodies for anti-rabbit IgG (H + L) and anti-mouse IgG (H + L) were obtained from Jackson ImmunoResearch (West Grove, PA). Endothelial cell medium was purchased from ScienCell Research Laboratories (Carlsbad, CA). RPMI–1640 culture medium, fetal bovine serum (FBS), and penicillin–streptomycin were bought from Gibco (Waltham, MA, USA). TMT10plex amino reactive reagents (0.8 mg per vial, #TF272312), radio immunoprecipitation assay (RIPA) buffer, silver stain for MS (#24600), and bicinchoninic acid (BCA) protein assay kit were purchased from Thermo Fisher Scientific (Waltham, MA, USA). Protease inhibitor (REF 05892791001) and phosphatase inhibitor (REF 04906837001) were acquired from Roche (Basel, Switzerland). Cell counting kit (CCK)–8 reagent was bought from Dojindo (Kumamoto, Japan). PrimeScript Master Mix and SYBR Premix Ex Taq were obtained from Takara (Shiga, Japan). All other reagents were either from Sigma Chemical Co. (St. Louis, MO) or Thermo Fisher Scientific (Waltham, MA, USA).

### Animal experiments

Male C57BL/6 J mice (8 weeks old, 20–22 g) were supplied by the Laboratory Animal Center of Shanghai University of Traditional Chinese Medicine (SHUTCM, Shanghai, China) and maintained on the standard laboratory by a 12-h light–dark cycle at 22 °C–25 °C and 55% ± 5% humidity-controlled environment with food and water ad libitum prior to the experiment. All animals were received humane care in compliance with regulations of experimental animal administration issued by the State Committee of Science and Technology of the People's Republic of China and the related ethics regulations of SHUTCM. All experiments were approved by the animal research committee of SHUTCM (Registration No. PZSHUTCM190628024 retrospectively registered at June 28, 2019). Prior to treatment, the mice were randomly divided into different groups, fasted overnight but with free access to water, and finally anesthetized with sodium pentobarbital for the collection of bio-samples.

Fifty-five mice were randomly divided into 5 groups with 11 members each and then orally treated with a single dose of 0.9% sodium chloride or senecionine (50 mg/kg body weight) for 1, 2, 12, and 24 h for toxicity evaluation. Eight mice from each group were anesthetized with sodium pentobarbital for the collection of blood samples and liver tissues. Serum alanine aminotransferase (ALT) and aspartate aminotransferase (AST) activities were determined with a Hitachi Automatic Analyzer 7080 (Hitachi High-Tech Science Systems Corp, Ibaraki, Japan). An aliquot of the liver tissues was applied for haematoxylin and eosin (HE) staining. The remaining three mice in each group were separately immobilized with glutaraldehyde through perfusion to prepare liver samples for scanning electron microscope (SEM), as described in our previous report (Zhang et al. 2019).

In addition, 12 mice were randomly divided into 3 groups with 4 members each and then orally treated with a single dose of 0.9% sodium chloride or senecionine (50 mg/kg body weight) for 2 and 12 h for the primary culture of mouse HSECs (mHSECs) by collagenase perfusion, metrizamide gradient centrifugation, and elutriation in accordance with the previous reported methods (Ruart et al. 2019; DeLeve et al. 2003). Total proteins and RNA were immediately extracted from the primary cultured mHSECs for further analysis.

To further delineate the pathogenic role of TSP1 in senecionine-induced HSOS, LSKL peptide (leucine-serine-lysine-leucine, a known TSP1 inhibitory peptide) was administered intraperitoneally (30 mg/kg bodyweight) at 0.25 and 6 h after senecionine treatment (50 mg/kg bodyweight). Mice were sacrificed at 12 h after senecionine treatment for sample collection.

### TMT-based shotgun proteomics study

The total proteins of mHSECs were prepared using pre-cold 8 M urea in 20 mM Tris–HCl buffer (pH 8.0) containing protease and phosphatase inhibitor. Protein samples for proteomics were randomly selected from nine mice, i.e., three mice from the VEH group, three mice from SEN–2 h group, and three mice from SEN–12 h. An aliquot of proteins (150 μg) from each sample was reduced by incubating with 10 mM Tris (2–carboxyethyl) phosphine (TCEP) buffer at 55 °C for 1 h and then alkylating with 18.75 mM iodoacetamide for 30 min at room temperature in the dark. Six volumes of pre-chilled acetone were added to the mixture to precipitate the proteins at  – 20 °C overnight. The protein pellet was then air-dried for 3 min, subsequently resuspended with 100 mM HEPES (pH 8.5), and digested with trypsin at 1:40 (w/w) overnight at 37 °C. The concentration of tryptic-digested peptide solution was measured with quantitative colorimetric peptide assay kit (Thermo Fisher Scientific, Waltham, MA, USA). An aliquot of peptides (80 μg in 0.1 mL) from each sample was mixed with TMT10plex amino reactive reagents (0.8 mg in 0.041 mL of anhydrous acetonitrile). All the solutions were mixed on a vortex. Reactions were allowed to proceed at room temperature for 1 h and then quenched by the addition of 0.008 mL of 5% hydroxylamine for 15 min at room temperature. The TMT-labeled samples were pooled into a new microcentrifuge tube at a 1:1:1:1:1:1:1:1:1 ratio. The mixture was centrifuged to dryness by Speedvac and desalted using a MonoSpin™ C18 column (GL Science, Tokyo, Japan).

Off-line fractionation was performed using an Agilent 1260 quaternary pump HPLC (Agilent Technologies, California, USA) and a Waters Acquity BEH C18 column (4.6 mm × 250 mm, 3.5 μm). The mobile phase consisted of buffer A (100% H_2_O), buffer B (100% acetonitrile), and buffer C (50 mM ammonium hydroxide, pH 10). A quarter of the pooled TMT-labeled peptides were resuspended in 300 μL of 10% buffer C. The elution time was 75 min with a 58 min gradient from 5 to 80% buffer B at a flow rate of 0.5 mL/min. Buffer C was constantly running at 10% of the total flow rate during the process. Fractions were collected within 75 min at 1.5 min intervals in 1.5 mL Eppendorf tubes collecting in rows. Finally, the peptide mixture was collected in a total of 50 tubes, which were consolidated into 25 fractions. The samples were subsequently centrifuged to dryness by Speedvac and redissolved in 5% formic acid for LC–MS/MS analysis.

All 25 fractions and peptide mixture of gel were analyzed by a homemade 30-cm-long pulled-tip analytical column (75 μm ID packed with ReproSil-Pur C18–AQ 1.9 μm resin, Dr. Maisch GmbH, Germany) placed in-line with an Easy-nLC 1200 nano HPLC (Thermo Scientific, San Jose, CA) for MS analysis. The analytical column temperature was set at 55 °C during the experiments. The mobile phase consisted of 0.1% formic acid in water (A) and 0.1% formic acid in 80% acetonitrile (B), and the elution gradient used for peptide separation was as follows: 0–1 min, 5–10% B; 1–96 min, 10–40% B; 96–104 min, 40–60% B, 104–105 min, 60–100% B, 105–120 min, 100% B. The flow rate was set at 300 nL/min.

Data-dependent tandem MS/MS analysis was performed with a Q Extractive Orbitrap mass spectrometer (Thermo Scientific, San Jose, CA). Peptides eluted from the column were directly electro-sprayed into the mass spectrometer with the application of a distal 2.2 kV spray voltage. A cycle of one full-scan MS spectrum (*m/z* 300–1800) was acquired, followed by the top 20 MS/MS events (TMT-labeled peptides were set the first mass fixed at 100 m*/z* scan range) sequentially generated on the first to the twentieth most intense ions selected from the full MS spectrum at a 28% normalized collision energy. Full scan resolution was set to 70,000 with automated gain control (AGC) target of 3 × e^^^6. MS/MS scan resolution was set at 17,500 (TMT-labeled peptides were set at 35,000) with an isolation window of 1.8 m*/z* and AGC target of 1 × e^^^5. The number of micro scans was one for both scans, and the maximum ion injection time was 50 and 100 ms for MS and MS/MS, respectively. The dynamic exclusion settings were as follows: charge exclusion, 1 and > 8; exclude isotopes, on; and exclusion duration, 30 s. MS scan functions and LC solvent gradients were controlled by the Xcalibur data system (Thermo Scientific, Waltham, MA, USA).

### Cell culture and treatments

Human hepatic sinusoidal endothelial cells (hHSECs) were bought from PriCells (Wuhan, Hubei, China) and cultured in endothelial cell medium supplemented with 5% FBS and 1% endothelial cell growth supplement (ECGS), 100 U/ml penicillin, and 100 mg/ml streptomycin, and kept in a humidified atmosphere at 37 °C and 5% CO_2_. And cells within six passages were used.

hHSECs were seeded into six-well plates at the density of 0.5–2 × 10^^^5 in a 0.2-mL volume and then incubated with solvent control or serial concentrations of senecionine (0.5–2.5 mM) for different time periods (24–72 h). After treatment, the cells were incubated with 10% CCK–8 in the culture medium for 2 h. Optical density was measured at 450 nm and cell viability was normalized as the percentage of solvent control.

In addition, hHSECs were seeded into six-well plates at the density of 0.5–2 × 10^^^5 in a 2-mL volume and then incubated with serial concentrations of senecionine (0.5–2 mM) for different time periods (24–72 h). After treatments, the protein lysate of cells was extracted with RIPA lysis buffer (0.2 mL per well) and applied for protein concentration assay using a BCA protein assay kit. An aliquot of the cell lysate (0.1 mL) was used for the quantification of PPAs by a pre-column derivatization-based LC–MS/MS approach following a previous method (Xiong et al. 2020). The PPAs content in cells was measured as nmol/g protein.

### Western blot

Protein samples were separated through SDS-PAGE gel electrophoresis, electrophoretically transferred onto PVDF membranes (Merck Millipore, Billerica, MA, USA), blocked with SuperBlock™ Blocking Buffer in PBS (Thermo Fisher Scientific, Waltham, MA, USA) for 2 h at room temperature, and washed with PBST (1% Tween–20 in PBS). The membranes were then subsequently incubated with the appropriate primary antibody at 4 °C overnight, followed by secondary HRP-conjugated antibody at room temperature for 2 h. Proteins in the membranes were visualized by Immobilon Western chemiluminescent HRP substrate (Merck Millipore, Billerica, MA, USA). β–Actin was used as the internal control.

### qPCR

Cellular total RNA was extracted using an RNA Faster200 reagent kit (Fastagen, Shanghai, China), and cDNA was synthesized by applying the PrimeScript™ RT Master Mix Kit (Takara, Shiga, Japan) in accordance with the manufacturer’s instructions. qPCR analysis was performed using the SYBR Premix Ex Taq™ Kit (Takara, Shiga, Japan). The relative expression of target genes was quantified using the 2^−ΔΔCt^ method and normalized to that of *Gapdh*. The primer sequences are shown in Table 1.

**Table 1:** Primers for qPCR analysis

Gene	Forward (5′ to 3′)	Reverse (5′ to 3′)
*mHSECs*		
*Gapdh*	CTGCTTCACCCCTCTCTTATT	GTGGCTCATCATCACACATT
*Icam1*	CCCCAACTCTTCTTGATGTATT	GATCTTTCCCCAGACTCTCAC
*Vcam1*	AGGGGACTGTCTGTCTGGGTTC	GTTGGGGATTCGGTTGTTCTG
*Cxcr4*	GACGACAGTCATCCTCATCCTA	CGAAGTCACATCCTTGCTTG
*Tnf-α*	GCTGAGCTCAAACCCTGGTA	CTCCAAAGTAGACCTGCCCG
*Thbs1*	GTGAGGTTTGTCTTTGGAACCA	GTTGTTGTCAAGGGTAAGAAGGA
*Mmp9*	ACTACGATAAGGACGGCAAAT	TCAAAGATGAACGGGAACAC
*Cd47*	TGCGGTTCAGCTCAACTACTG	ACGATGCAAGGGATGACCAC
*Tp53*	CTCTCCCCCGCAAAAGAAAAA	CGGAACATCTCGAAGCGTTTA
*Tgfb1*	AAGGACCTGGGTTGGAAGTG	CGGGTTGTGTTGGTTGTAGAG
*hHSECs*		
*GAPDH*	GGGAAGGTGAAGGTCGGAGT	GGGGTCATTGATGGCAACA
*ICAM1*	GGCATTGTCCTCAGTCAGAT	TCCTTCCTCTTGGCTTAGTCA
*NOS2*	GGGATGACTTTCCAAGACACA	CTGGGTCCTCTGGTCAAACTT
*CXCR4*	GAAATCATCAAGCAAGGGTGT	CAAGGAAAGCATAGAGGATGG
*MMP9*	GCTGGGCTTAGATCATTCCTC	ATTCACGTCGTCCTTATGCAA
*CD47*	TCCGGTGGTATGGATGAGAAA	ACCAAGGCCAGTAGCATTCTT
*TP53*	GAGGTTGGCTCTGACTGTACC	TCCGTCCCAGTAGATTACCAC

### Immunocytochemical staining

hHSECs were plated in 24-well plates at a density of 1–2 × 10^5 in the 500 μL of culture medium. After attachment, the cells were incubated with solvent control or serial concentrations of senecionine (0.5–2 mM) for different time periods (2–48 h), fixed with 4% paraformaldehyde for 20 min, permeabilized with 0.1% Triton X–100 for 5 min, and then blocked with 5% bovine serum albumin (BSA) in PBS for 1 h. The cells were then incubated with the anti-TSP1 antibody at 5 µg/mL overnight at 4 °C. The secondary antibodies were goat anti-rabbit IgG H&L (Alexa Fluor® 594) preadsorbed (colored red) and used at a 1:200 dilution for 1 h at room temperature. DAPI was employed to stain the cell nuclei (colored blue). Images for fluorescence immunostaining were examined under a confocal microscope (Olympics Fluoview FV–1000). Three independent experiments were performed.

Immunostaining of TSP1 was also conducted on the liver tissues of mice and humans, including patients with PA-induced HSOS (*n* = 3) and normal controls (*n* = 3). Human tissue materials were obtained from Nanjing Drum Tower Hospital, the Affiliated Hospital of Nanjing University School of Medicine (Jiangsu Province, China) in 2019. Signed informed consent was obtained from all participants prior to enrollment. This study was approved by the Ethics Committee of Nanjing Drum Tower Hospital and was designed and performed in accordance with the ethical guidelines of the 1975 Helsinki Declaration. All patients were diagnosed with PA-induced HSOS via consuming *G. japonica* in accordance with the previously described criteria (Zhuge et al. 2019).

### Immunoprecipitation assays

Pull-down assay was conducted in hHSECs and mouse liver using an anti-TSP1 antibody and an immunoprecipitation kit (Abcam, #ab206996, Cambridge, MA, USA) following the manufacturer’s protocol to verify the modification sites of TSP1 to dehydrosenecionine. hHSECs were plated in 100-mm plates at a density of 1 × 10^5 in 10 mL of culture medium and then treated with blank solvent or senecionine (1 mM) for 48 h. The cells were collected and lysed using a non-denaturing lysis buffer. An aliquot of the proteins (500 µg) was incubated overnight at 4 °C with intermittent shaking with 3 μg/mg rabbit control IgG (ABclonal, #AC005) or TSP1 rabbit polyclonal antibody (Abcam, #ab85762). The antigen–antibody complex was then mixed with pre-washed protein A/G Sepharose® beads and incubated for 1 h at 4 °C. At the same time, according to the same method, we extracted and purified TSP1 from the liver of mice after intragastric administration of senecionine (50 mg/kg) or 0.9% sodium chloride for 12 h in vivo. The antigen–antibody Sepharose bead complexes were washed and bound proteins eluted with SDS sample buffer heated at 95 °C of 5 min for SDS-PAGE (Bio-Rad) and silver-stained for MS following the manufacturer’s protocol (Thermo Fisher Scientific, Waltham, MA, USA). The TSP1-specific band around 129–155 kDa was cut and subjected to general in-gel pretreatment (Cao et al. 2016) and then identified by MS. Three independent experiments were performed.

### Data analysis

The LC–MS/MS data of TMT-labeled peptides of mHSECs were analyzed against a UniProtKB mouse (database released on 12th April, 2019) using Proteome Discoverer 2.1 (Thermo Scientific) with Sequest HT search engine. Precursor mass tolerance was set to ± 20 ppm, and fragment mass tolerance was set to ± 0.02 Da. The carbamidomethylation of cysteine (+ 57.021 Da) and TMT-labeled N-terminus and lysine (+ 229.163 Da) was set as static modification, and the oxidation of methionine (+ 15.995 Da) was set as dynamic modification. False discovery rate (FDR) for all identified proteins was set at 1% and calculated at the peptide level. The mass spectrometry proteomics data have been deposited to the ProteomeXchange Consortium (http://proteomecentral.proteomexchange.org) via the iProX partner repository (Ma et al. 2019) with the dataset identifier PXD026211. All the normalized reporter ion abundances of biological repeats were averaged for fold-change calculation. *T*-test was performed on each quantification ratio using a two-tailed one-sample *t*-test statistics module to obtain a *p* value. Proteins were considered to be differentially expressed at a *p* value cut-off of 0.05 and |log2 (fold change) |≥ 0.58.

Peptide identification and post-translational modification (PTM) analysis of gel from hHSECs and mouse livers were performed against a UniProtKB human (database released on 14^th^ August, 2020) using PEAKS Studio 8.5 software (Bioinformatics Solutions Inc., Waterloo, ON, CA) with Data Refinement, Auto De Novo, and Peaks Search. The mass tolerance of precursor ions and fragment ions was set to ± 20 ppm and ± 0.02 Da, respectively. Carbamidomethylation (+ 57.021 Da, C), oxidation (+ 15.995 Da, M), and DHP (+ 135.0684 Da, STKCDER) were set as variable modifications. The FDR for protein and peptides was set at a maximum of 1%. The identified proteins with at least one unique peptide and peptides with high average local confidence (ALC) ≥ 80% were accepted.

Function analyses for the differentially expressed proteins were performed according to their involvement in common biological processes through three different categories of gene ontology (GO): biological processes (BP), molecular functions (MF), and protein classes (PC). GO enrichment analysis was conducted using the PANTHER Classification System (http://pantherdb.org). Network analysis was performed by the STRING software (https://string-db.org/). The network generated by STRING was then reconstructed by Cytoscape Ver 3.8.1.

Other quantitative data, including serum biochemical values, Western blot quantitative results, and mRNA quantitative findings, were expressed as the mean value ± standard error of the mean value (SEM). Data were analyzed by the Student’s *t*-test or one-way ANOVA analysis with LSD post hoc test using GraphPad Prism (Version 8; GraphPad, La Jolla, CA, USA).*p* < 0.05 was considered as statistically significant. Hierarchical cluster analysis (HCA) and heatmap were conducted using an online tool powered by R language (http://www.omicsolution.org/wu-kong-beta-linux/main/).

## Results

### Senecionine induces severe liver injury in mice with initial damage in the hepatic sinusoid

HSOS models in rodents have been established by oral exposure to monocrotaline (a type of PAs commonly found in herbs/plants from *Fabaceae* family) (DeLeve et al. 1999; Nakamura et al. 2002; Huang et al. 2019) and to senecionine (one of the major PAs present in *G. japonica*) (Lin et al. 2011; Yang et al. 2017; Hessel-Pras et al. 2020). In the last decades, PA-induced HSOS cases are often associated with the oral intake of *G. japonica* (Lin et al. 2011; Zhuge et al. 2019). Therefore, we here induced a previously reported murine model of senecionine-induced HSOS by exposing C57BL/6 J male mice to a single dose of senecionine (50 mg/kg body weight) (Yang et al. 2017). And the time-course of the model was described in our present study.

Compared with the naïve control (the VEH group), serum ALT and AST activities initially increased at 12 h in the murine HSOS model induced by senecionine and further increased at 24 h (Fig. 1). HE staining showed inflammatory cell infiltration in the hepatic lobules of mice treated with senecionine for 12 h. Severe liver injuries, including hepatic sinusoidal hemostasis, endothelial damage to the central vein, and coagulative necrosis of hepatocytes, emerged in the mice treated with senecionine for 24 h. SEM images showed the loss of fenestrae (defenestration) organized as sieve plates in the sinusoidal of mice treated with senecionine for 1 h. This injury aggravated throughout the investigated time and included the formation of large gaps in the sinusoidal endothelium, the exposure of hepatocytes, and the visibility of hepatocyte microvilli through those gaps (Fig. 1d). Therefore, senecionine induces severe liver damage in mice with initial damage to the hepatic sinusoid.

### Senecionine causes dynamic changes in the proteome of mHSECs isolated from mice with HSOS

In the rat HSOS model induced by single oral administration of 160 mg/kg monocrotaline (DeLeve et al. 1999 and 2003), a description of the time-course of the experimental model suggested that days 0.5 through 2 constitute a pre-HSOS phase with loss of HSECs fenestrae and formation of gaps within and between HSECs. The yield of HSECs isolated from the model rats on day 1 (the pre-HSOS phase) after monocrotaline was comparable with the normal rats (DeLeve et al. 2003). The expression of CD31 on the cell surface is regarded as a marker for defenestrated HSECs and was observed in the isolated HSECs from rats with thioacetamide-induced liver fibrosis despite extensive digestion (DeLeve et al. 2004). In our present study, we also isolated HSECs from mice with HSOS induced by single oral administration of 50 mg/kg senecionine and found no obvious change in the yield of mHSECs between the model mice (on 2 and 12 h after senecionine exposure) and the normal mice (SI Fig. 1). After attachment, the mHSECs isolated from normal mice and HSOS mice all showed a typical cobblestone, sheet-like appearance, and positive expression of CD31. Notably, CD31 was expressed in the cytoplasm of HSECs from normal mice yet more HSECs from HSOS mice (on 12 h after senecionine exposure) showed CD31 expression on the borders of HSECs (SI Fig. 1), implying defenestration of HSECs in mice treated with senecionine. These data confirmed the successful isolation of HSECs from normal and HSOS mice.

Most PAs are metabolically activated to form PPAs that initiate toxicity (Ma et al. 2018; Fu et al. 2004). The existence of PPAs has been reported in the liver, blood, microsomes, and different cell lines upon PA treatment. Therefore, PPAs have been recommended as the xenobiotic biomarkers for PA-induced toxicity. We also quantified the levels of PPAs in the isolated mHSECs by an LC–MS/MS approach as previous described (Xiong et al. 2020). PPAs level was ~ 60 nmol/g protein in mHSECs from mice with HSOS (on 12 h after senecionine exposure) (SI Fig. 1), implying that mHSECs were undoubtably injured by senecionine exposure. Thus, the mHSECs were then subjected to shotgun proteomics study by tandem mass tagged (TMT–10plex) LC–MS/MS analysis. Among the 7143 identified proteins from mHSECs, 7117 proteins were quantified. The threshold of ratios was set at 1.5-fold (|log2 (fold change) |≥ 0.58) in TMT data analyses to identify the differentially expressed proteins in mHSECs related to senecionine-induced toxicity.

**Fig. 2:**
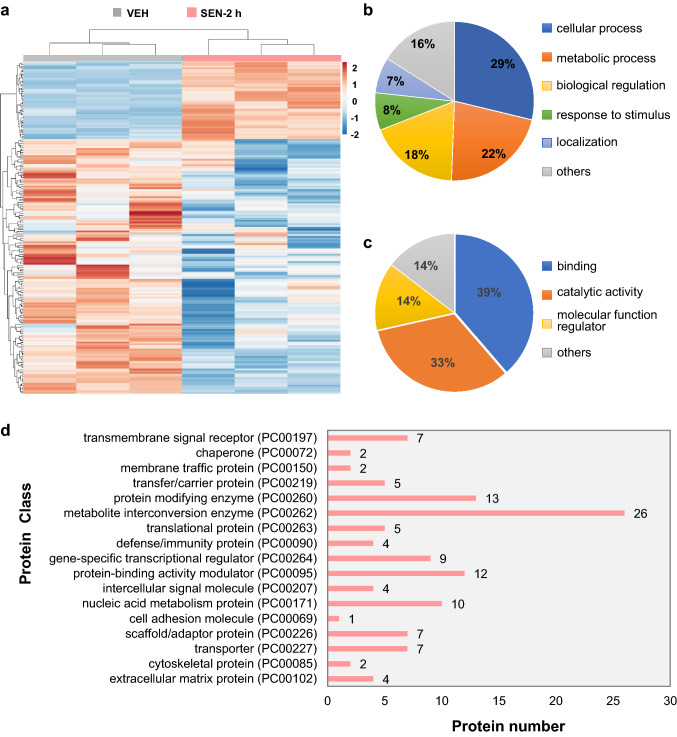
Proteome changes in mice upon senecionine treatment (50 mg/kg body weight) for 2 h (*n* = 3). **a** Tree diagram of HCA of the differentially expressed proteins. Differential expressed proteins were screened by a *p* value ≤ 0.05 and |i.e., log2(fold change)|≥ 0.58. **b** Biological processes. **c** Molecular functions. d Protein classification

A total of 142 down-regulated and 43 up-regulated proteins were found in the mice treated with senecionine for 2 h (Fig. 2a and SI Table 1). BP analysis suggested that the altered proteins were mainly related to the cellular process (GO: 0,009,987), metabolic process (GO: 0,008,152), biological regulation (GO: 0,065,007), response to stimulus (GO: 0,050,896), and localization (GO: 0,051,179) (Fig. 2b). MF analysis showed that most of the differentially expressed proteins were enriched in catalytic activity (GO: 0,003,824), including hydrolase, oxidoreductase, and transferase activity, followed by binding (GO: 0,005,488), and molecular function regulator (GO: 0,098,772) (Fig. 2c). In addition, many differentially expressed proteins were classified to metabolite inter conversion enzyme (PC00262), protein modifying enzyme (PC00260), protein-binding activity modulator (PC00095), nucleic acid metabolism protein (PC00171) and gene-specific transcriptional regulator (PC00264) (Fig. 2d).

**Fig. 3:**
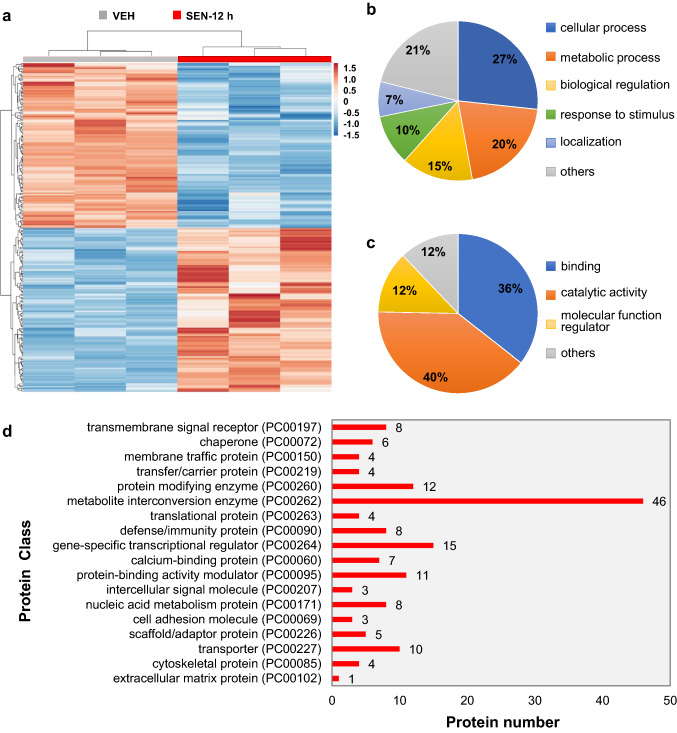
Proteome changes in mice upon senecionine treatment (50 mg/kg body weight) for 12 h (*n* = 3). **a** Tree diagram of HCA of the differentially expressed proteins. Differential expressed proteins were screened by a *p* value ≤ 0.05 and |i.e., log2(fold change)|≥ 0.58. **b** Biological processes. **c** Molecular functions. d Protein classification

Besides, a total of 115 up-regulated and 116 down-regulated proteins were observed in the mice treated with senecionine for 12 h (Fig. 3a and SI Table 2). Results from BP analysis (Fig. 3b) and MF analysis (Fig. 3c) of the altered proteins in SEN–12 h groups showed general consistency with that in SEN–2 h group. However, more proteins in metabolite interconversion enzyme (PC00262) were identified in SEN–12 h group (46 proteins) than SEN–2 h group (26 proteins). Besides, 7 calcium-binding proteins (PC00060) were additionally identified in SEN–12 h groups (Fig. 3d). These data suggested that senecionine causes dynamic changes in the proteome of mHSECs.

### TSP1 overexpression is a contributor to senecionine-induced HSOS

**Fig. 4:**
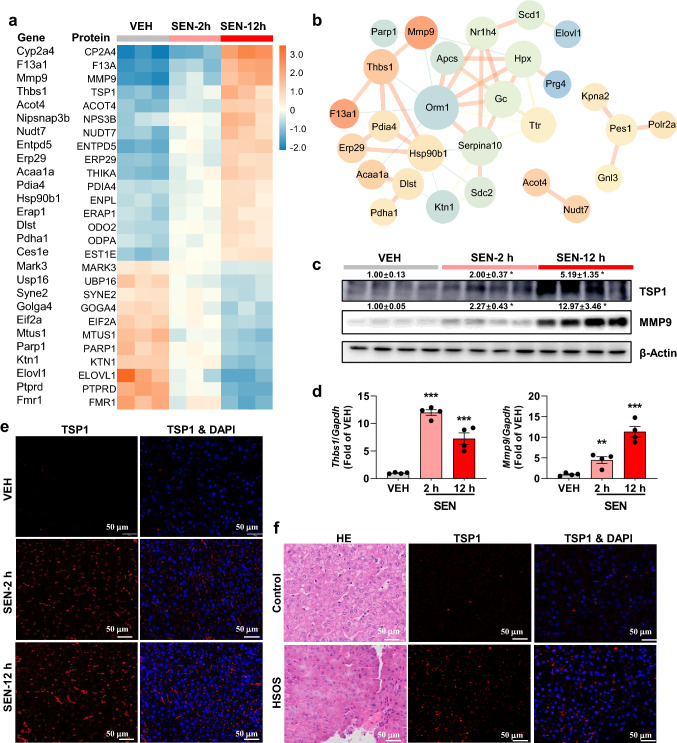
TSP1 contributes to senecionine-induced toxicity. Proteins significantly changed in all group pairs were selected and visualized by heatmap (**a**) and PPI network (**b**). The network was drawn by String and reconstructed by Cytoscape software. Size of the node represents the fold change (SEN-12 h *vs.* VEH) of the protein while thickness of the edge indicates the Person correlation. Red means up-regulated or positive connected while blue means down-regulated or negative connected. The protein (**c**) and mRNA (**d**) expression of TSP1 (*Thbs1*) and MMP9 (*Mmp9*) in mHSECs (*n* = 4). Values are expressed as mean ± SEM. *p < 0.05, ***p < 0.001 *vs.* VEH group. **e** Representative immunologically staining of liver tissues in mice (*n* = 3). f Representative HE and immunocytochemical staining of liver tissues in control and patients with HSOS (*n* = 3). Liver tissues are stained with TSP1 in red and DAPI in blue. Scale bar: 50 µm

Among all the quantified proteins, 50 proteins were found to be significantly changed in all group pairs, i.e., VEH vs SEN–2 h, VEH vs SEN–12 h, and SEN–2 h vs SEN–12 h (SI Table 3). And 16 proteins were increased with exposure time while 11 proteins were decreased with exposure time (Fig. 4a). A protein–protein interaction (PPI) network was constructed and suggested the important of TSP1 in senecionine-induced toxicity (Fig. 4b). TSP1 was overexpressed upon senecionine treatment and strong positive connection (> 0.9) was found between TSP1 with several proteins including MMP9 (Mmp9, Matrix metalloproteinase-9), F13A (F13a1, Coagulation factor XIII A chain), PDIA4 (Pdia4, Protein disulfide-isomerase A4), and ENPL (Hsp90b1, Endoplasmin). Notably, high score was found for the interaction between TSP1 with MMP9 (score 0.965), a well-recognized ligand for TSP1 (Bein and Simons 2000) and a recognized marker for HSOS (DeLeve et al. 2003).

TSP1 is a matricellular glycoprotein that mediates cell-to-cell and cell-to-matrix interactions (Rogers et al. 2017; Shetty et al. 2018). Through binding to extracellular proteins and/or cell surface receptors, TSP1 plays important roles in multiple biological processes including coagulation, cell adhesion, cell growth, modulation of cell–cell and cell–matrix interactions, control of tumor growth and metastases, and angiogenesis (Resovi et al. 2014). We then performed Western blot of the primary cultured mHSECs from mice in the VEH and model groups. Compared with those in the VEH mice, the protein levels of TSP1 and MMP9 were up-regulated in mHSECs upon senecionine treatment, especially in the mice treated with senecionine for 12 h (increased by 5.19- and 12.97-fold, respectively) (Fig. 4c). The mRNA expression levels of *Thbs1* and *Mmp9* were also up-regulated in the mHSECs upon senecionine treatment (Fig. 4d). In addition, CD47 (Integrin associated protein, IAP), another receptor of TSP1, was also up-regulated in mice treated with senecionine (SI Fig. 2). TSP1 and p4N1 (i.e., the CD47-binding fragment of TSP1) can dose-dependently induce HSEC defenestration (Venkatraman and Tucker-Kellogg 2013) thus to progress and regress hepatic fibrosis (Xie et al. 2012). Our previous study also proved that PAs induce HSOS in mice via upregulating fibrosis-related factors, i.e., TGFβ/SMAD and inflammatory signaling (Zhang et al. 2019). TSP1 can activate TGFβ to promote fibrosis via triggering several intracellular signaling pathways to regulate downstream profibrotic molecules (Schultz-Cherry et al. 1994; Breitkopf et al. 2005). The protein and mRNA expression levels of TGFB1 were increased in mHSECs from mice treated with senecionine in the present study (SI Fig. 2), which supports the above reports. What’s more, TSP1 also acts as an essential component of the inflammatory response. Inflammation has been associated with the pathogenesis of HSOS induced by monocrotaline (Zhang et al. 2017; Huang et al. 2019) and herbal extracts containing PAs (Zhang et al. 2019). TSP1 can up-regulate the adhesion proteins ICAM-1 and VCAM-1 on endothelial cells through CD47 (Narizhneva et al. 2005), and this finding was consistent with the present data that senecionine triggered the inflammatory response in HSECs as verified by the overexpression of adhesion molecules and chemokines in mHSECs (SI Fig. 2).

Considering the importance of TSP1 in senecionine-induced toxicity, the expression of TSP1 upon senecionine treatment was also visualized by immunocytochemical staining. As results, TSP1 was time-dependently over-expressed in the liver of mice with senecionine treatment (Fig. 4e). What is more, an increased TSP1 expression was also observed in the liver of patients with PA-induced HSOS (Fig. 4f). The mRNA expression levels of *MMP9*, *CD47*, *TP53*, as well as adhesion molecules and chemokines such as *ICAM-1*, also increased in hHSECs (SI Fig. 3). These results suggest that overexpression of TSP1 (and other related molecules) upon senecionine treatment is converted from mice to humans and may further impair cellular function and trigger toxicity. Therefore, blocking TSP1 might be a new therapy for PA-induced HSOS in clinics.

**Fig. 5:**
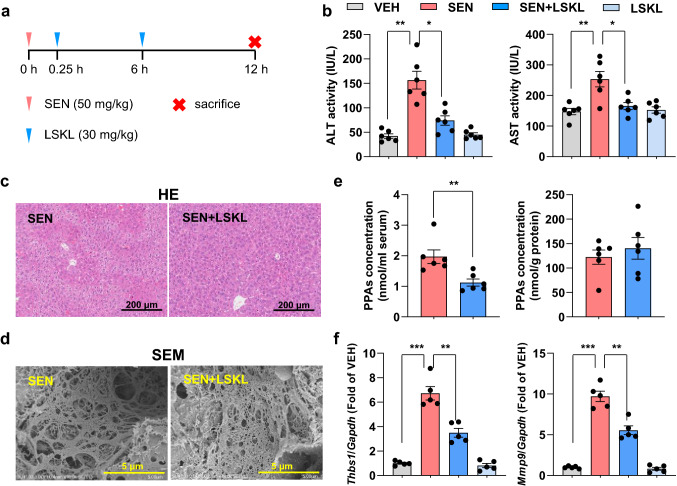
TSP1 inhibitory LSKL peptide protects from senecionine-induced HSOS in mice. **a** Mice were administered intraperitoneally with LSKL peptide (at 30 mg/kg body weight) at 0.25 and 6 h after senecionine exposure (50 mg/kg body weight) and sacrificed at 12 h. **b** Serum ALT and AST activities (*n* = 6). Values are expressed as mean ± SEM. *p < 0.05, **p < 0.01. **c** Typical images of HE staining of liver tissues (*n* = 3). Scale bar: 200 µm. **d** Typical scanning electron microscopy images of liver tissues (*n* = 3). Scale bar: 5 µm. **e** Contents of PPAs in serum and liver samples (*n* = 6). Values are expressed as mean ± SEM. **p < 0.01. **f** The mRNA expression of *Thbs1* and *Mmp9* in mouse livers (*n* = 5). Values are expressed as mean ± SEM. **p < 0.01, ***p < 0.001

LSKL is a known TSP1 inhibitor (Kondou et al. 2003; Kuroki et al. 2015). Only two doses of LSKL peptide during the early period after hepatectomy (30 mg/kg bodyweight administered intraperitoneally before abdominal wall closure and at 6 h after the 70% hepatectomy) can promote liver regeneration in mice (Kuroki et al. 2015). We used LSKL to test whether pharmacological inhibition of TSP1 can protect mice from senecionine-induced toxicity. The magnitude of increase in serum ALT and AST activities was decreased in mice that were treated with LSKL after senecionine exposure (Fig. 5). The extent of senecionine-induced hepatic parenchymal cell injury and the damage in the sinusoidal endothelium (evident by loss of fenestration on the sieve plate and the formation of large gaps in the sinusoidal endothelium) was also reduced in mice that were treated with LSKL. Additionally, the serum levels of PPAs, the xenobiotic marker for PA-induced toxicity, decreased by 43% (*p *< 0.01) in mice that were treated with LSKL after model induction (Fig. 5e) and the magnitude of the increase in the mRNA expression of *Thbs1* and *Mmp9* in disserted liver tissues was reduced by LSKL treatment (Fig. 5f). These results demonstrate that inhibition of TSP1 with the LSKL peptide can confer therapeutic effect against senecionine-induced HSOS in mice.

### TSP1 is covalently modified by the reactive pyrrole generated from senecionine in hHSECs

**Fig. 6:**
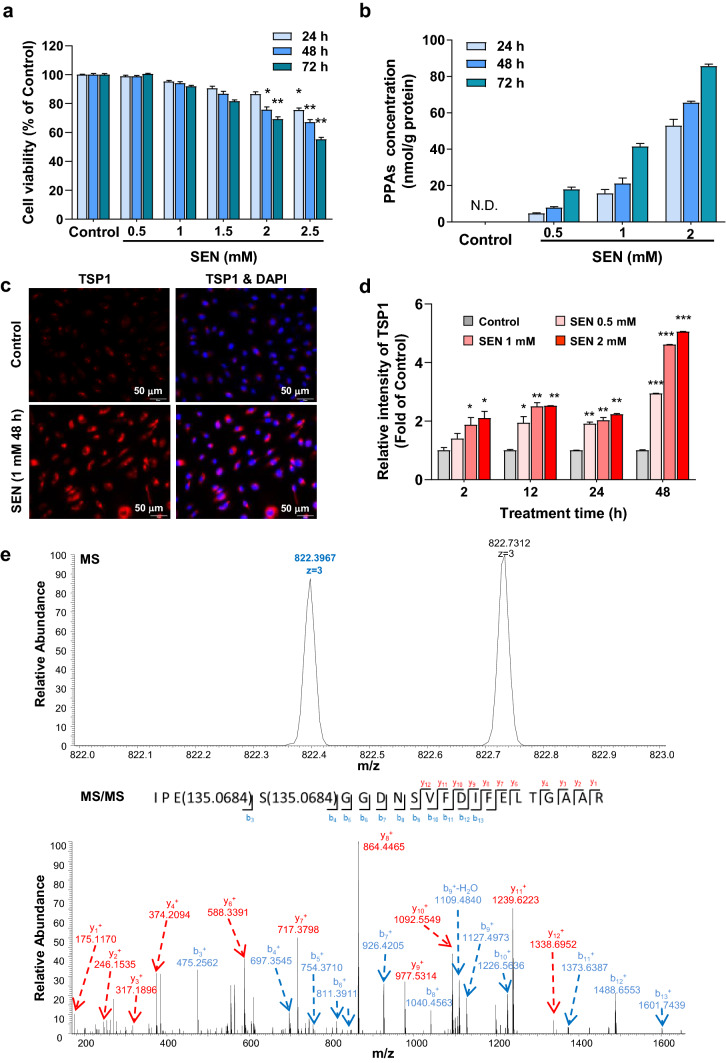
TSP1 is covalently modified by DHP in hHSECs upon senecionine treatment. hHSECs were incubated with blank solvent (control) or senecionine (0.5–2.5 mM) for different time periods. Three independent experiments were performed. **a** Cell viability (*n* = 8). **b** Contents of PPAs in hHSECs (*n* = 3). Values are expressed as mean ± SEM. *p < 0.05, **p < 0.01, ***p < 0.001 *vs.* control group. **c** Representative immunocytochemical staining images of hHSECs. hHSECs are stained with TSP1 in red and DAPI in blue. Scale bar: 50 µm. **d** Relative intensity of TSP1 in hHSECs by immunocytochemical staining (*n* = 3). Values are expressed as mean ± SEM. *p < 0.05, **p < 0.01, ***p < 0.001 *vs.* control group. **e** MS spectrum showing the potential modification sites of DHP (135.0684 Da) in TSP1 in hHSECs after senecionine treatment (1 mM) for 48 h. The upper panel shows the MS spectrum of human TSP1. The lower panel shows a higher energy collision-induced dissociation (HCD) MS/MS spectrum recorded on the [M + 3H]^3+^ ion at m/z 822.3967 of the human TSP1 peptide IPESGGDNSVFDIFELTGAAR harboring two DHP site. Predicted b- and y-type ions (not including all) are listed below and above the peptide sequence, respectively. Matched ions are labeled in the spectrum and indicate that TSP1 is modified on E23 and S24

Most of PAs are metabolized by CYP450s to form the reactive pyrrole of dehydropyrrolizidine alkaloids, which interact rapidly with cellular proteins to generate PPAs that trigger liver injury (Fu et al. 2004; Lin et al. 2011). In this study, senecionine dose- and time-dependently induced cytotoxicity in hHSECs (Fig. 6a). After senecionine treatment, the contents of PPAs also increased with dose and incubation time in hHSECs (Fig. 6b). In the present study, PPAs contents were 5–60 nmol/g protein in the primary hHSECs treated with 0.5–2 mM senecionine for 24 h. PPAs contents were ~ 60 nmol/g protein in HSECs from mice with HSOS (on 12 h after senecionine exposure) (SI Fig. 1). Therefore, senecionine induced toxicity in vivo and in vitro at comparable doses.

TSP1 overexpression in hHSECs upon senecionine treatment was confirmed by immunocytochemical staining (SI Fig. 4). TSP1 expression was increased with dose and incubation time upon senecionine treatment in hHSECs (Fig. 6d). This finding is consistent with the results in mHSECs. Whether and how TSP1 is modified by the reactive pyrrole remain unknown. hHSECs were then incubated with senecionine for the extraction and purification of TSP1 using a pull-down strategy (Wiktorowicz et al. 2019). MS analysis discovered two potential DHP residues (135.0684 Da), namely, SER–24 (S24) and GLU–23 (E23) in TSP1 (Fig. 6e). Therefore, TSP1 is covalently bonded to the reactive pyrrolic metabolites of senecionine to form the TSP1–DHP adducts in hHSECs. We also extracted and purified TSP1 from disserted liver tissue of mice treated with senecionine (50 mg/kg bodyweight) for 12 h. As expected, the same adducts of DHP modification at the S24 and E23 sites were also found in mice (SI Fig. 5). These data indicate that senecionine is metabolically activated to form dehydrosenecionine, which further binds with TSP1 to further impair cellular function and trigger toxicity.

## Discussion

HSOS is a rare hepatic vascular disease with high mortality and diverse causes. PA exposure via herbal medicines, healthy supplementary, and contaminated food is one of the main causes of HSOS world-wide. PA-induced HSOS cases have markedly increased, and *G. japonica* is currently the leading cause (Chen et al. 2007; Zhuge et al. 2019). However, no effective treatment is available in clinic. The earliest manifestations of PA-induced HSOS are the progressive injury to the sinusoidal wall with loss of sinusoidal lining cells, sinusoidal hemorrhage, and mild damage to central vein endothelium (DeLeve et al. 1999; Zhuge et al. 2019). Therefore, the initial and crucial pathogenic event of PA-induced HSOS occurs in HSECs.

Most of PAs are metabolically activated by liver CYP450s to form PPAs that initiate toxicity (Ma et al. 2018). Physiologically, HSECs have rather low abundance of active CYP450s, leading to the low efficiency to metabolize PAs by HSECs. Monocrotaline treatment (4 mM for 16 h) caused a 40% decrease in cell viability of freshly isolated rat HSECs (DeLeve et al. 2003) and PPAs was ~ 8 nmol/g protein in hHSECs treated with monocrotaline (300 µM for 48 h) (Yang et al. 2016). In our present study, senecionine treatment (2.5 mM for 72 h) caused a 45% decreased in cell viability of hHSECs and PPAs at ~ 90 nmol/g protein. We also reported a 25% decrease in cell viability and PPAs at ~ 600 nmol/g protein in primary cultured mouse hepatocytes by senecionine treatment (10 µM for 48 h) (Xiong et al. 2020). These data proved that hepatocytes are more efficiency in PA metabolism; yet PAs can at least partly be metabolized by HSECs to trigger cytotoxicity in HSECs.

Recently, new HSECs models such as the two-layer transwell co-culture model with HSECs and HepaRG (Lu et al. 2019) and the CYP3A4-transduced HSECs (Lu et al. 2020) are developed for the toxicity evaluation of PAs. In these models, PAs are metabolic activated by CYP450s in HepaRG or by CYP3A4 transduced into HSECs thus to trigger toxicity. In our present study, mice were orally treated with senecionine (50 mg/kg body weight), which can be metabolized by hepatocytes in mice liver. Then HSECs were isolated from the mice. PPAs level in HSECs from mice with HSOS (on 12 h after senecionine exposure) were ~ 60 nmol/g protein and is comparable to that in the whole lysis of liver tissues. These data suggested that senecionine was metabolic activated in liver (mainly by hepatocytes) and then trigger damages to HSECs. Thus, the HSECs, where the initial and crucial pathogenic event of PA-induced HSOS occurs, were isolated from mice and applied for a TMT-based shotgun proteomics study in our present study.

As results, dramatic and time-dependent changes were uncovered in the proteome of mHSECs from mice with HSOS (on 2 and 12 h after senecionine exposure). Most of the differentially expressed proteins upon senecionine treatment in mHSECs were involved in the cellular and metabolic processes. In addition, the majority of differentially expressed proteins were rich in catalytic activity, such as hydrolase and enzyme regulator activities. These findings were consistent with the widely accepted concept that PAs are metabolically activated to generate their toxicity, which involves metabolic enzymes such as liver CYP450s, flavin-containing monooxygenase, esterase (Fu et al. 2004). PPAs contain an identical core pyrrole moiety regardless of the structures of PAs; however, the proteins that can form PPAs are largely unknown. Lamé et al. (2000 and 2005) suggested that the exclusive conjugation of proteins in the cytosol is associated with the membranes in contact with the cytosol (plasma membrane, endoplasmic reticulum, and mitochondria) in human pulmonary artery endothelial cells upon dehydromonocrotaline treatment. Proteins containing the CYS-X-X-CYS motif are the most remarkably modified owing to their subcellular locations and reactive thiol groups. Pyrrole-ATP synthase subunit beta adduct was identified in retrosine-treated rats and human cell lines, as a cause of impaired mitochondrial function (Lu et al. 2018). In the present study, two potential pyrrole residues in TSP1, which is mainly located in the cytoplasm and extracellular matrix and contains abundant thiol groups in the protein structure, were novelty detected in hHSECs and mouse livers.

TSP1 is involved in many liver diseases such as liver fibrosis (Li et al. 2017). TSP1 is also reported to exacerbate azoxymethane-induced acute liver failure by activating TGFB1 signaling (Jefferson et al. 2020). In present study, strong interaction between TSP1 with MMP9 upon senecionine treatment was uncovered by PPI analysis. MMP9 is a well-recognized ligand of TSP1 (Bein and Simons, 2000). The interaction of them have been associated with various wound healing processes, such as haemostasis, inflammation, proliferation, and remodeling. In present study, over-expressed TSP1 and MMP9 upon senecionine treatment were observed both in vivo and in vitro. The earliest manifestations of HSOS (0.5 to 2 days after model induction) were progressive injury to the sinusoidal wall with loss of sinusoidal lining cells and an early increase of MMP9, as well as a later lower-magnitude increase of MMP2 (DeLeve et al. 1999 and 2003). And HSECs were identified as the major source of both basal and monocrotaline-induced MMP9 among liver cells including hepatocytes, stellate cells, and Kupffer cells (DeLeve et al. 2003). MMP9 has also been recognized as the therapeutic target of experimental HSOS in rodents (DeLeve et al. 2003; Periasamy et al. 2011; Nakamura et al. 2012). Thus, there is a strong connection between the overexpression of TSP1/MMP9 and PA-induced HSOS. In our present study, LSKL peptide, the known TSP1 inhibitor, protected mice against senecionine-induced HSOS. As expected, contents of PPAs (xenobiotic markers for PA-induced HSOS) and expression of MMP9 (a well-recognized marker for HSOS) were also reduced in mice that were treated with two doses of LSKL peptide during the early period after model induction. These results demonstrate that inhibition of TSP1 with the LSKL peptide confer a therapeutic effect against senecionine-induced HSOS in mice.

To the authors’ knowledge, this work is the first TMT-based shotgun proteomics study in HSECs isolated from mice with HSOS (induced by senecionine). Dynamic changes in the proteome of mHSECs occurred at the initial period of damage after model induction. And TSP1 played a central role, which may be associated with pathologies such defenestration of HSECs, inflammation, and fibrosis by interacting with its ligands. TSP1 inhibitory LSKL peptide treatment during the early period after model induction can protect from HSOS in mice. What is more, TSP1 over expression upon PA treatment was converted from mice to humans and pyrrole-TSP1 adduct was novelty identified in hHSECs treated with senecionine and livers from mice exposed to senecionine, suggesting that TSP1 may serve as a potential biomarker in investigating the pathogenesis of PA-induced HSOS. This study deciphers new molecular mechanisms for this disease and lays the scientific basis for the clinical treatment of PA-induced HSOS.

## Supplementary Information

Below is the link to the electronic supplementary material.

Supplementary file1 (DOCX 83 KB)

Supplementary file2 (PDF 755 KB)

## Data Availability

The authors declare that the data supporting the findings of this study are available within the paper. All other data are available from the corresponding author upon reasonable request.

## Abbreviations

ALT: Alanine aminotransferase
AST: Aspartate aminotransferase
CYP450s: Cytochrome P450 enzymes
HSECs: Hepatic sinusoidal endothelial cells
HSOS: Hepatic sinusoidal obstruction syndrome
MMP9: Matrix metallopeptidase 9
PA: Pyrrolizidine alkaloid
PPAs: Pyrrole-protein adducts
TSP1: Thrombospondin 1
