# Supplementary material for: A TMT-based shotgun proteomics uncovers overexpression of thrombospondin 1 as a contributor in pyrrolizidine alkaloid-induced hepatic sinusoidal obstruction syndrome
Source: Arch Toxicol. 2022 Mar 31;96(7):2003–19. doi: 10.1007/s00204-022-03281-7 (PMC9151551; doi:10.1007/s00204-022-03281-7)
Supplement: Supplementary file 1 — Supplementary file1 (DOCX 83 KB) [file 204_2022_3281_MOESM1_ESM.docx]

**Supplementary Information**

**A TMT-based shotgun proteomics uncovers** **overexpression of thrombospondin 1 as a contributor in pyrrolizidine alkaloid-induced hepatic sinusoidal obstruction syndrome**

Weiqian Wang ^#^, Yan Chen ^#^, Yue Yin, Xunjiang Wang, Xuanling Ye, Kaiyuan Jiang, Yi Zhang, Jiwei Zhang, Wei Zhang, Yuzheng Zhuge, Li Chen, Chao Peng^*^, Aizhen Xiong^*^, Li Yang^*^, Zhengtao Wang

# Weiqian Wang and Yan Chen are contributed equally to this work.

* Address correspondence to: yl7@shutcm.edu.cn (Li Yang); a.z.xiong@hotmail.com (Aizhen Xiong); pengchao@sari.ac.cn (Chao Peng).

**SI Table 1.** Differentially expressed proteins between mice treated with blank solvent and senecionine for 2 h.

| **Uniprot ID** | **Protein** | ***p*-value** | **log2 Fold change (SEN-2h/VEH)** |
| --- | --- | --- | --- |
| *Up-regulated proteins upon senecionine treatment* | | | |
| Q8BJ56 | PNPLA2 | 0.0051 | 2.14 |
| Q60765 | ATF3 | 0.0333 | 2.06 |
| O54791 | MAFF | 0.0201 | 1.73 |
| P09926 | SURF2 | 0.0039 | 1.50 |
| Q9CX48 | ZCCHC10 | 0.0356 | 1.42 |
| Q3U1Z5 | GPSM3 | 0.0031 | 1.30 |
| P28563 | DUSP1 | 0.0349 | 1.29 |
| Q8VEH0 | TMEM144 | 0.0209 | 1.29 |
| P39689 | CDKN1A | 0.0083 | 1.27 |
| Q8CDJ8 | STON1 | 0.0342 | 1.22 |
| Q9CSV6 | SFT2D3 | 0.0216 | 1.20 |
| O88543 | COPS3 | 0.0295 | 1.13 |
| P24815 | HSD3B1 | 0.0114 | 1.12 |
| Q7TQI7 | ABTB2 | 0.0078 | 1.11 |
| Q9CZ62 | CEP97 | 0.0030 | 1.10 |
| Q61585 | G0S2 | 0.0302 | 1.08 |
| Q9WUA5 | EPM2A | 0.0217 | 1.00 |
| P23950 | ZFP36L1 | 0.0004 | 0.99 |
| Q9DCJ9 | NPL | 0.0368 | 0.98 |
| Q05928 | BTC | 0.0195 | 0.95 |
| P43883 | PLIN2 | 0.0013 | 0.93 |
| Q8VDH1 | FBXO21 | 0.0188 | 0.87 |
| P00416 | MT-CO3 | 0.0096 | 0.85 |
| Q8C0L9 | GPCPD1 | 0.0349 | 0.85 |
| Q9Z2X2 | PSMD10 | 0.0254 | 0.82 |
| Q6IS41 | SLC25A47 | 0.0044 | 0.81 |
| Q9DBX2 | PDCL | 0.0005 | 0.80 |
| Q91YL3 | UCKL1 | 0.0257 | 0.80 |
| Q9CXC3 | MGME1 | 0.0136 | 0.78 |
| Q8BWU8 | ETNPPL | 0.0012 | 0.77 |
| P52840 | SULT1A1 | 0.0092 | 0.71 |
| O09043 | NAPSA | 0.0453 | 0.71 |
| Q91X44 | GCKR | 0.0048 | 0.68 |
| Q9CQL0 | METTL21A | 0.0244 | 0.68 |
| Q3TIU4 | PDE12 | 0.0156 | 0.67 |
| P24547 | IMPDH2 | 0.0240 | 0.67 |
| Q9ERI5 | JMJD6 | 0.0032 | 0.66 |
| Q14DH7 | ACSS3 | 0.0223 | 0.66 |
| P21550 | ENO3 | 0.0385 | 0.64 |
| P01867 | IGH-3 | 0.0292 | 0.63 |
| Q8BVE3 | ATP6V1H | 0.0423 | 0.62 |
| Q99MK9 | RASSF1 | 0.0497 | 0.60 |
| Q91XD6 | VPS36 | 0.0229 | 0.59 |
| *Down-regulated proteins upon senecionine treatment* | | | |
| P48437 | PROX1 | 0.0128 | -0.58 |
| P22361 | HNF1A | 0.0439 | -0.58 |
| P63254 | CRIP1 | 0.0077 | -0.59 |
| O35730 | RING1 | 0.0405 | -0.59 |
| Q9R0Q9 | MPDU1 | 0.0069 | -0.59 |
| Q91WV7 | SLC3A1 | 0.0019 | -0.59 |
| Q6A098 | SECISBP2L | 0.0111 | -0.59 |
| P62492 | RAB11A | 0.0058 | -0.59 |
| O89051 | ITM2B | 0.0108 | -0.60 |
| Q9QUJ7 | ACSL4 | 0.0070 | -0.60 |
| Q91XD7 | CRELD1 | 0.0066 | -0.60 |
| Q9D1J3 | SARNP | 0.0437 | -0.60 |
| Q99L28 | RSL24D1 | 0.0483 | -0.61 |
| P01029 | C4B | 0.0111 | -0.61 |
| Q99PJ0 | NTM | 0.0039 | -0.61 |
| Q61207 | PSAP | 0.0081 | -0.62 |
| P47964 | RPL36 | 0.0047 | -0.62 |
| Q9CR41 | HYPK | 0.0448 | -0.62 |
| Q7TQE6 | MACO1 | 0.0009 | -0.62 |
| Q9JKW0 | ARL6IP1 | 0.0270 | -0.62 |
| Q9CQS9 | HAUS2 | 0.0322 | -0.62 |
| Q6PAQ4 | REXO4 | 0.0062 | -0.62 |
| Q9D0M5 | DYNLL2 | 0.0117 | -0.63 |
| Q924W5 | SMC6 | 0.0299 | -0.63 |
| Q9CYA0 | CRELD2 | 0.0257 | -0.63 |
| Q9DBE9 | FTSJ3 | 0.0171 | -0.63 |
| Q8C8T8 | TSR2 | 0.0039 | -0.63 |
| P52019 | SQLE | 0.0087 | -0.64 |
| Q99JG7 | TNIP2 | 0.0156 | -0.64 |
| Q6ZWU9 | RPS27 | 0.0065 | -0.64 |
| Q9EPL0 | XYLT2 | 0.0418 | -0.64 |
| Q8BWT5 | DIP2A | 0.0168 | -0.64 |
| Q9D198 | SYF2 | 0.0408 | -0.65 |
| Q8R121 | SERPINA10 | 0.0079 | -0.65 |
| Q80Z37 | TOPORS | 0.0008 | -0.65 |
| Q640L3 | CCPG1 | 0.0184 | -0.66 |
| Q9R022 | DNAJC12 | 0.0033 | -0.66 |
| Q9CR96 | TMEM208 | 0.0106 | -0.66 |
| Q8BWW4 | LARP4 | 0.0016 | -0.66 |
| Q9Z131 | SH3BP5 | 0.0050 | -0.66 |
| Q8R0K4 | CCDC137 | 0.0028 | -0.66 |
| P07309 | TTR | 0.0345 | -0.67 |
| Q9JJT0 | RCL1 | 0.0009 | -0.67 |
| P53996 | CNBP | 0.0192 | -0.67 |
| Q61704 | ITIH3 | 0.0372 | -0.67 |
| Q9JLJ5 | ELOVL1 | 0.0388 | -0.68 |
| Q91VE6 | NIFK | 0.0179 | -0.68 |
| P19001 | KRT19 | 0.0018 | -0.68 |
| Q8C767 | PPP1R3B | 0.0075 | -0.69 |
| Q99JF8 | PSIP1 | 0.0374 | -0.69 |
| Q9D1R9 | RPL34 | 0.0020 | -0.69 |
| Q69ZS0 | PDZRN3 | 0.0050 | -0.70 |
| Q08501 | PRLR | 0.0027 | -0.70 |
| P31532 | SAA4 | 0.0152 | -0.70 |
| O54904 | B3GALT1 | 0.0280 | -0.70 |
| Q9CPT5 | NOP16 | 0.0494 | -0.71 |
| Q03734 | SERPINA3M | 0.0006 | -0.71 |
| P43274 | H1-4 | 0.0053 | -0.71 |
| Q8K003 | TMA7 | 0.0054 | -0.72 |
| P06684 | C5 | 0.0027 | -0.72 |
| O35143 | ATP5IF1 | 0.0464 | -0.72 |
| Q9D8N6 | LIN37 | 0.0106 | -0.72 |
| Q9Z2M6 | UBL3 | 0.0090 | -0.72 |
| O35245 | PKD2 | 0.0460 | -0.72 |
| A2A995 | FYB2 | 0.0244 | -0.73 |
| P12246 | APCS | 0.0014 | -0.73 |
| Q0VG62 | RBIS | 0.0082 | -0.73 |
| Q00560 | IL6ST | 0.0123 | -0.73 |
| Q9DB94 | WDR53 | 0.0311 | -0.73 |
| Q60775 | ELF1 | 0.0016 | -0.73 |
| Q8BGC3 | SLC16A12 | 0.0204 | -0.74 |
| P49817 | CAV1 | 0.0032 | -0.75 |
| Q60641 | NR1H4 | 0.0130 | -0.75 |
| P55088 | AQP4 | 0.0099 | -0.75 |
| Q9CRA4 | MSMO1 | 0.0017 | -0.75 |
| Q01237 | HMGCR | 0.0005 | -0.75 |
| Q9R1Q6 | TMEM176B | 0.0178 | -0.76 |
| Q9CY57 | CHTOP | 0.0088 | -0.76 |
| P01027 | C3 | 0.0002 | -0.76 |
| Q9CZJ1 | UTP11 | 0.0004 | -0.77 |
| P01592 | JCHAIN | 0.0133 | -0.78 |
| Q8BGD9 | EIF4B | 0.0230 | -0.78 |
| P39061 | COL18A1 | 0.0075 | -0.78 |
| Q9WTN3 | SREBF1 | 0.0209 | -0.78 |
| Q71KU9 | FGL1 | 0.0004 | -0.79 |
| Q9CQ92 | FIS1 | 0.0040 | -0.79 |
| Q9JLZ6 | HIC2 | 0.0310 | -0.80 |
| Q920B0 | FRMD4B | 0.0026 | -0.80 |
| A2AL36 | CNTRL | 0.0062 | -0.81 |
| P40224 | CXCL12 | 0.0018 | -0.81 |
| Q9DA19 | CIR1 | 0.0083 | -0.81 |
| P13516 | SCD1 | 0.0035 | -0.81 |
| P21614 | GC | 0.0106 | -0.82 |
| Q923B6 | STEAP4 | 0.0052 | -0.82 |
| Q8R516 | MIB2 | 0.0098 | -0.83 |
| Q01339 | APOH | 0.0229 | -0.83 |
| Q7TPZ8 | CPA1 | 0.0042 | -0.85 |
| P70275 | SEMA3E | 0.0380 | -0.85 |
| Q75N73 | SLC39A14 | 0.0082 | -0.86 |
| O08580 | ESRRA | 0.0337 | -0.86 |
| P06909 | CFH | 0.0227 | -0.86 |
| Q9EQQ2 | YIPF5 | 0.0056 | -0.86 |
| O35988 | SDC4 | 0.0063 | -0.87 |
| P34928 | APOC1 | 0.0060 | -0.89 |
| Q9DCS1 | TMEM176A | 0.0019 | -0.90 |
| Q9Z1L3 | DEDD | 0.0398 | -0.91 |
| Q64285 | CEL | 0.0261 | -0.95 |
| Q9JHH6 | CPB2 | 0.0094 | -0.97 |
| Q9D4H9 | PHF14 | 0.0226 | -1.01 |
| Q62179 | SEMA4B | 0.0034 | -1.02 |
| O09030 | IL13RA1 | 0.0467 | -1.04 |
| P47879 | IGFBP4 | 0.0230 | -1.05 |
| P16882 | GHR | 0.0207 | -1.06 |
| Q78YY6 | DNAJC15 | 0.0275 | -1.07 |
| E9Q414 | APOB | 0.0005 | -1.07 |
| Q61646 | HP | 0.0054 | -1.07 |
| P56656 | CYP2C39 | 0.0309 | -1.08 |
| P43407 | SDC2 | 0.0072 | -1.09 |
| Q3UVU3 | SLC30A10 | 0.0335 | -1.09 |
| Q7TNS2 | MICOS10 | 0.0130 | -1.12 |
| Q91WP6 | SERPINA3N | 0.0000 | -1.14 |
| P53798 | FDFT1 | 0.0079 | -1.15 |
| Q9QXQ1 | PDE7B | 0.0014 | -1.18 |
| P03899 | MTND3 | 0.0089 | -1.19 |
| Q8BFQ4 | WDR82 | 0.0078 | -1.20 |
| O70570 | PIGR | 0.0011 | -1.21 |
| Q80W65 | PCSK9 | 0.0167 | -1.23 |
| P28798 | GRN | 0.0036 | -1.32 |
| Q8BKU8 | TMEM87B | 0.0364 | -1.32 |
| Q9CTG6 | ATP13A2 | 0.0103 | -1.33 |
| P07361 | ORM2 | 0.0125 | -1.37 |
| Q60590 | ORM1 | 0.0003 | -1.53 |
| Q60997 | DMBT1 | 0.0012 | -1.68 |
| Q9CR35 | CTRB1 | 0.0061 | -1.78 |
| Q9JM99 | PRG4 | 0.0021 | -1.92 |
| Q9CQW5 | LGALS2 | 0.0229 | -2.12 |
| Q9Z1R3 | APOM | 0.0130 | -2.15 |
| P00688 | AMY2 | 0.0020 | -2.17 |
| D3Z6P0 | PDIA2 | 0.0116 | -2.19 |
| P05366 | SAA1 | 0.0020 | -2.32 |
| Q8K0C5 | ZG16 | 0.0004 | -2.38 |
| P05367 | SAA2 | 0.0025 | -2.83 |

The threshold of fold change (FC) was set at 1.5-fold (i.e., |log2 (FC) | ≥ 0.58).

**SI Table 2.** Differentially expressed proteins between mice treated with blank solvent and senecionine for 12 h.

| **UniProt ID** | **Protein** | ***p*-value** | **log2 Fold change (SEN-12h/VEH)** |
| --- | --- | --- | --- |
| *Up-regulated upon senecionine treatment* | | | |
| Q60765 | ATF3 | 0.0016 | 1.98 |
| P51437 | CAMP | 0.0245 | 1.77 |
| O54791 | MAFF | 0.0048 | 1.60 |
| P09450 | JUNB | 0.0095 | 1.55 |
| P39689 | CDKN1A | 0.0055 | 1.46 |
| O08692 | NGP | 0.0012 | 1.46 |
| Q8BJ56 | PNPLA2 | 0.0341 | 1.44 |
| P08071 | LTF | 0.0089 | 1.43 |
| P15392 | CYP2A4 | 0.0001 | 1.37 |
| Q8R2S8 | CD177 | 0.0360 | 1.35 |
| P47955 | RPLP1 | 0.0140 | 1.28 |
| O35744 | CHIL3 | 0.0049 | 1.20 |
| Q4VGL6 | RC3H1 | 0.0463 | 1.16 |
| P05555 | ITGAM | 0.0264 | 1.14 |
| P41245 | MMP9 | 0.0013 | 1.14 |
| P08905 | LYZ2 | 0.0000 | 1.14 |
| P11247 | MPO | 0.0264 | 1.12 |
| Q8BH61 | F13A1 | 0.0008 | 1.12 |
| P07091 | S100A4 | 0.0066 | 1.11 |
| P04202 | TGFB1 | 0.0376 | 1.10 |
| P61939 | SERPINA7 | 0.0195 | 1.10 |
| Q8CIH5 | PLCG2 | 0.0042 | 1.06 |
| P24547 | IMPDH2 | 0.0268 | 1.04 |
| O89053 | CORO1A | 0.0028 | 1.03 |
| B2RS91 | RRN3 | 0.0008 | 1.02 |
| Q8K558 | TREML1 | 0.0191 | 1.01 |
| P01869 | IGHG1 | 0.0061 | 1.01 |
| Q03145 | EPHA2 | 0.0001 | 1.00 |
| O70324 | SLC16A2 | 0.0411 | 1.00 |
| Q91WK5 | GCSH | 0.0196 | 0.99 |
| P32037 | SLC2A3 | 0.0029 | 0.96 |
| O55071 | CYP2B19 | 0.0257 | 0.95 |
| P28293 | CTSG | 0.0002 | 0.94 |
| O08970 | TUFT1 | 0.0110 | 0.89 |
| Q9QX60 | DGUOK | 0.0227 | 0.89 |
| E2JF22 | PIEZO1 | 0.0095 | 0.88 |
| Q9QYR7 | ACOT3 | 0.0089 | 0.87 |
| P97501 | FMO3 | 0.0157 | 0.86 |
| P13745 | GSTA1 | 0.0173 | 0.86 |
| Q9Z2F6 | BCL3 | 0.0180 | 0.85 |
| Q6P069 | SRI | 0.0259 | 0.83 |
| P15037 | ETS2 | 0.0056 | 0.82 |
| Q00612 | G6PDX | 0.0470 | 0.82 |
| P10107 | ANXA1 | 0.0139 | 0.81 |
| Q9QYI6 | DNAJB9 | 0.0041 | 0.79 |
| P97369 | NCF4 | 0.0402 | 0.79 |
| Q61827 | MAFK | 0.0240 | 0.79 |
| Q8CBB9 | RSAD2 | 0.0168 | 0.78 |
| Q9Z0F4 | CIB1 | 0.0348 | 0.78 |
| P43883 | PLIN2 | 0.0287 | 0.77 |
| P02088 | HBB-B1 | 0.0243 | 0.77 |
| Q64518 | ATP2A3 | 0.0093 | 0.76 |
| Q9WUZ9 | ENTPD5 | 0.0003 | 0.76 |
| Q9CQR4 | ACOT13 | 0.0039 | 0.75 |
| P56404 | AQP8 | 0.0249 | 0.75 |
| Q8K4F5 | ABHD11 | 0.0244 | 0.75 |
| P02340 | TP53 | 0.0081 | 0.74 |
| O88466 | ZNF106 | 0.0011 | 0.74 |
| P00330 | ADH1 | 0.0007 | 0.74 |
| Q64267 | XPA | 0.0435 | 0.74 |
| Q9CZS1 | ALDH1B1 | 0.0003 | 0.73 |
| P35441 | THBS1 | 0.0254 | 0.73 |
| P20108 | PRDX3 | 0.0070 | 0.72 |
| B2RPV6 | MMRN1 | 0.0013 | 0.72 |
| P41317 | MBL2 | 0.0010 | 0.71 |
| Q9D6H2 | HSPB11 | 0.0266 | 0.70 |
| Q3UV74 | ITGB2L | 0.0486 | 0.70 |
| Q76KJ5 | POLR1G | 0.0267 | 0.70 |
| Q99J99 | MPST | 0.0114 | 0.70 |
| Q91WU0 | CES1F | 0.0014 | 0.69 |
| Q3TP92 | CTDNEP1 | 0.0072 | 0.68 |
| Q9CWQ0 | DPH5 | 0.0062 | 0.68 |
| Q6NTA4 | RRAGB | 0.0256 | 0.67 |
| Q8BWN8 | ACOT4 | 0.0008 | 0.67 |
| P31725 | S100A9 | 0.0335 | 0.67 |
| Q3UV55 | NR1D1 | 0.0004 | 0.67 |
| Q923B1 | DBR1 | 0.0397 | 0.67 |
| P13634 | CA1 | 0.0319 | 0.66 |
| Q99MK9 | RASSF1 | 0.0223 | 0.66 |
| Q3V3R1 | MTHFD1L | 0.0029 | 0.66 |
| P01942 | HBA | 0.0221 | 0.65 |
| Q9D924 | ISCA1 | 0.0149 | 0.65 |
| Q99KR7 | PPIF | 0.0163 | 0.65 |
| Q9D404 | OXSM | 0.0035 | 0.65 |
| Q921H8 | ACAA1A | 0.0029 | 0.65 |
| Q9D0B5 | TSTD3 | 0.0086 | 0.64 |
| O09110 | MAP2K3 | 0.0035 | 0.63 |
| Q91YY4 | ATPAF2 | 0.0044 | 0.63 |
| Q9DCJ9 | NPL | 0.0344 | 0.63 |
| Q8VCH0 | ACAA1B | 0.0074 | 0.62 |
| Q3TC72 | FAHD2 | 0.0004 | 0.62 |
| Q9QZA0 | CA5B | 0.0066 | 0.62 |
| Q9QYR9 | ACOT2 | 0.0213 | 0.62 |
| Q9DCU9 | HOGA1 | 0.0104 | 0.62 |
| P15327 | BPGM | 0.0156 | 0.62 |
| P57759 | ERP29 | 0.0011 | 0.62 |
| Q99MV1 | TDRD1 | 0.0097 | 0.62 |
| Q9CPV3 | MRPL42 | 0.0336 | 0.62 |
| Q64339 | ISG15 | 0.0120 | 0.61 |
| Q9WTQ5 | AKAP12 | 0.0053 | 0.61 |
| Q5FW57 | GM4952 | 0.0213 | 0.61 |
| Q8K327 | CHAMP1 | 0.0364 | 0.61 |
| Q8BZQ7 | ANAPC2 | 0.0316 | 0.60 |
| P23589 | CA5A | 0.0127 | 0.60 |
| P20852 | CYP2A5 | 0.0001 | 0.60 |
| Q3TFK5 | GPATCH4 | 0.0287 | 0.60 |
| Q9Z2I8 | SUCLG2 | 0.0086 | 0.59 |
| Q3U0Y2 | TMEM35B | 0.0027 | 0.59 |
| P14069 | S100A6 | 0.0317 | 0.59 |
| Q8CGK3 | LONP1 | 0.0031 | 0.59 |
| Q99LP6 | GRPEL1 | 0.0095 | 0.58 |
| Q9D665 | SDR42E1 | 0.0145 | 0.58 |
| P00920 | CA2 | 0.0297 | 0.58 |
| Q80VP0 | TECPR1 | 0.0143 | 0.58 |
| Q99P30 | NUDT7 | 0.0410 | 0.58 |
| *Down-regulated upon senecionine treatment* | | | |
| Q99PJ0 | NTM | 0.0201 | -0.58 |
| P01592 | JCHAIN | 0.0283 | -0.58 |
| Q80WR5 |  | 0.0148 | -0.58 |
| Q71KU9 | FGL1 | 0.0450 | -0.59 |
| Q99JF8 | PSIP1 | 0.0194 | -0.59 |
| Q8C8T8 | TSR2 | 0.0066 | -0.59 |
| Q91WE6 | CDKAL1 | 0.0006 | -0.59 |
| Q9CRA4 | MSMO1 | 0.0003 | -0.59 |
| Q0VG62 | RBIS | 0.0093 | -0.59 |
| O89086 | RBM3 | 0.0336 | -0.59 |
| Q61646 | HP | 0.0008 | -0.59 |
| Q63961 | ENG | 0.0006 | -0.59 |
| P31532 | SAA4 | 0.0085 | -0.60 |
| P43025 | CLEC3B | 0.0069 | -0.60 |
| Q03734 | SERPINA3M | 0.0005 | -0.60 |
| Q9Z1D1 | EIF3G | 0.0039 | -0.60 |
| Q78PY7 | SND1 | 0.0101 | -0.60 |
| Q8BGC3 | SLC16A12 | 0.0006 | -0.60 |
| Q9DB94 | WDR53 | 0.0456 | -0.61 |
| P31651 | SLC6A12 | 0.0332 | -0.61 |
| O35988 | SDC4 | 0.0083 | -0.62 |
| Q8C767 | PPP1R3B | 0.0037 | -0.62 |
| O70200 | AIF1 | 0.0207 | -0.62 |
| Q9R022 | DNAJC12 | 0.0081 | -0.62 |
| Q99PL5 | RRBP1 | 0.0028 | -0.62 |
| Q9CQ92 | FIS1 | 0.0041 | -0.62 |
| Q3TXT3 | INIP | 0.0049 | -0.63 |
| Q3V2Q8 | N4BP2L1 | 0.0269 | -0.63 |
| Q91Z49 | FYTTD1 | 0.0268 | -0.63 |
| Q61595 | KTN1 | 0.0017 | -0.63 |
| Q60953 | PML | 0.0003 | -0.63 |
| P20918 | PLG | 0.0046 | -0.63 |
| P62492 | RAB11A | 0.0293 | -0.63 |
| Q9D1J3 | SARNP | 0.0003 | -0.63 |
| O88735 | MAP7 | 0.0002 | -0.64 |
| Q6AW69 | CGNL1 | 0.0016 | -0.64 |
| P22361 | HNF1A | 0.0306 | -0.64 |
| Q922Q8 | LRRC59 | 0.0201 | -0.65 |
| Q61703 | ITIH2 | 0.0117 | -0.65 |
| E9PYL2 | PRR12 | 0.0317 | -0.65 |
| Q8BMD5 | CDADC1 | 0.0005 | -0.65 |
| Q02780 | NFIA | 0.0124 | -0.65 |
| Q01279 | EGFR | 0.0047 | -0.65 |
| E9Q6J5 | BOD1L | 0.0117 | -0.66 |
| P11103 | PARP1 | 0.0020 | -0.66 |
| P29341 | PABPC1 | 0.0205 | -0.66 |
| Q8R1S9 | SLC38A4 | 0.0070 | -0.66 |
| P97464 | EXT1 | 0.0141 | -0.66 |
| Q60590 | ORM1 | 0.0011 | -0.67 |
| Q8R4G6 | MGAT5 | 0.0152 | -0.67 |
| Q66JW3 | TOX | 0.0048 | -0.67 |
| O55003 | BNIP3 | 0.0025 | -0.67 |
| Q1ERP8 | CD300LG | 0.0024 | -0.68 |
| Q5HZI1 | MTUS1 | 0.0006 | -0.68 |
| Q91VJ2 | CAVIN3 | 0.0119 | -0.68 |
| Q8BFQ4 | WDR82 | 0.0195 | -0.69 |
| Q3UBG2 | PID1 | 0.0044 | -0.69 |
| P01027 | C3 | 0.0001 | -0.70 |
| Q9Z280 | PLD1 | 0.0020 | -0.70 |
| Q8R001 | MAPRE2 | 0.0251 | -0.70 |
| Q8R420 | ABCA3 | 0.0004 | -0.71 |
| Q00560 | IL6ST | 0.0211 | -0.72 |
| P28798 | GRN | 0.0141 | -0.73 |
| Q91ZX7 | LRP1 | 0.0000 | -0.73 |
| O89032 | SH3PXD2A | 0.0085 | -0.73 |
| P06684 | C5 | 0.0084 | -0.73 |
| Q01237 | HMGCR | 0.0014 | -0.74 |
| Q7TQE6 | MACO1 | 0.0011 | -0.75 |
| Q08501 | PRLR | 0.0044 | -0.75 |
| Q9WTN3 | SREBF1 | 0.0207 | -0.76 |
| Q8R1S4 | MTSS1 | 0.0003 | -0.76 |
| P28700 | RXRA | 0.0144 | -0.78 |
| Q80XU3 | NUCKS1 | 0.0031 | -0.78 |
| P30275 | CKMT1 | 0.0132 | -0.79 |
| Q61704 | ITIH3 | 0.0024 | -0.80 |
| Q9JLZ6 | HIC2 | 0.0005 | -0.80 |
| Q75N73 | SLC39A14 | 0.0191 | -0.81 |
| P01029 | C4B | 0.0000 | -0.82 |
| Q3UVU3 | SLC30A10 | 0.0457 | -0.83 |
| Q6NWW9 | FNDC3B | 0.0030 | -0.83 |
| Q7TNS2 | MICOS10 | 0.0339 | -0.84 |
| Q7TPZ8 | CPA1 | 0.0037 | -0.84 |
| Q8BRV5 | KIAA1671 | 0.0019 | -0.86 |
| Q8VDJ3 | HDLBP | 0.0048 | -0.87 |
| P97333 | NRP1 | 0.0118 | -0.88 |
| P40224 | CXCL12 | 0.0011 | -0.89 |
| O54904 | B3GALT1 | 0.0082 | -0.91 |
| P97360 | ETV6 | 0.0035 | -0.93 |
| Q9CPU9 | SLC31A2 | 0.0464 | -0.94 |
| Q2VLH6 | CD163 | 0.0068 | -0.94 |
| Q9CY57 | CHTOP | 0.0032 | -0.96 |
| Q8BLY1 | SMOC1 | 0.0014 | -0.97 |
| E9Q414 | APOB | 0.0002 | -0.98 |
| P06909 | CFH | 0.0192 | -0.98 |
| P35922 | FMR1 | 0.0005 | -1.00 |
| Q64487 | PTPRD | 0.0000 | -1.01 |
| Q9D4H9 | PHF14 | 0.0157 | -1.06 |
| Q64285 | CEL | 0.0233 | -1.07 |
| Q91WP6 | SERPINA3N | 0.0001 | -1.07 |
| P70194 | CLEC4F | 0.0000 | -1.09 |
| Q8BKU8 | TMEM87B | 0.0295 | -1.12 |
| Q9JLJ5 | ELOVL1 | 0.0183 | -1.17 |
| O70361 | PER3 | 0.0276 | -1.21 |
| Q9JM93 | ARL6IP4 | 0.0060 | -1.25 |
| Q61263 | SOAT1 | 0.0122 | -1.29 |
| P16882 | GHR | 0.0140 | -1.29 |
| Q9JM99 | PRG4 | 0.0063 | -1.34 |
| O70570 | PIGR | 0.0001 | -1.39 |
| Q05685 | FOLR2 | 0.0034 | -1.43 |
| Q9CR35 | CTRB1 | 0.0055 | -1.93 |
| Q60997 | DMBT1 | 0.0001 | -1.97 |
| Q8K0C5 | ZG16 | 0.0004 | -2.23 |
| P00688 | AMY2 | 0.0039 | -2.24 |
| P05366 | SAA1 | 0.0022 | -2.34 |
| D3Z6P0 | PDIA2 | 0.0081 | -2.70 |
| P05367 | SAA2 | 0.0024 | -3.03 |

The threshold of fold change was set at 1.5-fold (i.e., |log2 (FC) | ≥ 0.58).

**SI Table 3.** Significantly altered proteins between all group pairs.

| **UniProt ID** | **Protein** | **Gene** | **Name** | **log2 Fold change (SEN-2h/VEH)** | **log2 Fold change (SEN-12h/VEH)** | **log2 Fold change (SEN-12h/SEN-2h)** |
| --- | --- | --- | --- | --- | --- | --- |
| ***Increased with time upon senecionine treatment*** | | | | | | |
| P15392 | CP2A4 | Cyp2a4 | Cytochrome P450 2A4 | 0.34 * | 1.40 *** | 1.06 *** |
| Q8BH61 | F13A | F13a1 | Coagulation factor XIII A chain | 0.35 * | 1.13 ** | 0.77 *** |
| P41245 | MMP9 | Mmp9 | Matrix metalloproteinase-9 | 0.50 * | 1.14** | 0.64 ** |
| P35441 | TSP1 | Thbs1 | Thrombospondin-1 | 0.30 ** | 0.75 * | 0.44 * |
| Q8BWN8 | ACOT4 | Acot4 | Peroxisomal succinyl-coenzyme A thioesterase | 0.26 ** | 0.66 *** | 0.40 *** |
| Q9CQE1 | NPS3B | Nipsnap3b | Protein NipSnap homolog 3B | 0.19 * | 0.55 * | 0.35 * |
| Q99P30 | NUDT7 | Nudt7 | Peroxisomal coenzyme A diphosphatase NUDT7 | 0.29 * | 0.64 ** | 0.35 * |
| Q9WUZ9 | ENTPD5 | Entpd5 | Ectonucleoside triphosphate diphosphohydrolase 5 | 0.46 ** | 0.77 *** | 0.31 ** |
| P57759 | ERP29 | Erp29 | Endoplasmic reticulum resident protein 29 | 0.27 ** | 0.58 *** | 0.31 *** |
| Q921H8 | THIKA | Acaa1a | 3-ketoacyl-CoA thiolase A, peroxisomal | 0.38 * | 0.66 *** | 0.28 ** |
| P08003 | PDIA4 | Pdia4 | Protein disulfide-isomerase A4 | 0.15 * | 0.37 ** | 0.22 ** |
| P08113 | ENPL | Hsp90b1 | Endoplasmin | 0.17 * | 0.37 ** | 0.20 * |
| Q9EQH2 | ERAP1 | Erap1 | Endoplasmic reticulum aminopeptidase 1 | 0.18 * | 0.38 ** | 0.20 * |
| Q9D2G2 | ODO2 | Dlst | Dihydrolipoyllysine-residue succinyltransferase component of 2-oxoglutarate dehydrogenase complex, mitochondrial | 0.21 *** | 0.39 ** | 0.18 * |
| P35486 | ODPA | Pdha1 | Pyruvate dehydrogenase E1 component subunit alpha, somatic form, mitochondrial | 0.17 * | 0.35 ** | 0.18 ** |
| Q64176 | EST1E | Ces1e | Carboxylesterase 1E | 0.32 *** | 0.50 *** | 0.17 ** |
| ***Decreased with time upon senecionine treatment*** | | | | | | |
| Q03141 | MARK3 | Mark3 | MAP/microtubule affinity-regulating kinase 3 | -0.14 ** | -0.26 ** | -0.11 * |
| Q99LG0 | UBP16 | Usp16 | Ubiquitin carboxyl-terminal hydrolase 16 | -0.35 * | -0.47 ** | -0.12 * |
| Q6ZWQ0 | SYNE2 | Syne2 | Nesprin-2 | -0.16 ** | -0.34 *** | -0.19 ** |
| Q91VW5 | GOGA4 | Golga4 | Golgin subfamily A member 4 | -0.23 * | -0.46 ** | -0.24 * |
| Q8BJW6 | EIF2A | Eif2a | Eukaryotic translation initiation factor 2A | -0.27 ** | -0.51 ** | -0.24 ** |
| Q5HZI1 | MTUS1 | Mtus1 | Microtubule-associated tumor suppressor 1 homolog | -0.45 *** | -0.70 *** | -0.25 ** |
| P11103 | PARP1 | Parp1 | Poly [ADP-ribose] polymerase 1 | -0.31 * | -0.61 ** | -0.31 *** |
| Q61595 | KTN1 | Ktn1 | Kinectin | -0.28 ** | -0.65 *** | -0.37 ** |
| Q9JLJ5 | ELOVL1 | Elovl1 | Elongation of very long chain fatty acids protein 1 | -0.68 * | -1.06 * | -0.39 * |
| Q64487 | PTPRD | Ptprd | Receptor-type tyrosine-protein phosphatase delta | -0.52 *** | -0.97 *** | -0.45 *** |
| P35922 | FMR1 | Fmr1 | Synaptic functional regulator FMR1 | -0.43 ** | -0.91 ** | -0.49 * |
| ***Others*** | | | | | | |
| P07309 | TTHY | Ttr | Transthyretin | -0.67 * | 0.26 * | 0.93 ** |
| Q60590 | ORM1 | Orm1 | Alpha-1-acid glycoprotein 1 | -1.53 *** | -0.80 *** | 0.73 ** |
| P43407 | SDC2 | Sdc2 | Syndecan-2 | -1.09 ** | -0.36 ** | 0.72 * |
| P52293 | IMA1 | Kpna2 | Importin subunit alpha-1 | -0.24 ** | 0.34 *** | 0.58 *** |
| Q9WTV6 | UBP18 | Usp18 | Ubl carboxyl-terminal hydrolase 18 | -0.21 * | 0.36 ** | 0.57 ** |
| P21614 | GC | Gc | Vitamin D-binding protein | -0.82 * | -0.26 * | 0.55 * |
| Q8CI11 | GNL3 | Gnl3 | Guanine nucleotide-binding protein-like 3 | -0.31 * | 0.24 * | 0.55 ** |
| P08775 | RPB1 | Polr2a | DNA-directed RNA polymerase II subunit RPB1 | -0.18 * | 0.37 ** | 0.55 *** |
| Q9JM99 | PRG4 | Prg4 | Proteoglycan 4 | -1.92 ** | -1.40 ** | 0.52 * |
| P13516 | SCD1 | Scd1 | Acyl-CoA desaturase 1 | -0.81 ** | -0.31 * | 0.51 * |
| Q9EQ61 | PESC | Pes1 | Pescadillo homolog | -0.23 * | 0.26 ** | 0.49 *** |
| Q60641 | NR1H4 | Nr1h4 | Bile acid receptor | -0.75 * | -0.26 ** | 0.49 * |
| Q9CZJ1 | UTP11 | Utp11 | Probable U3 small nucleolar RNA-associated protein 11 | -0.77 *** | -0.38 * | 0.38 * |
| Q91WV7 | SLC3A1 | Slc3a1 | Neutral and basic amino acid transport protein rBAT | -0.59 ** | -0.25 * | 0.34 * |
| Q8R121 | ZPI | Serpina10 | Protein Z-dependent protease inhibitor | -0.65 ** | -0.33 * | 0.32 * |
| Q91X72 | HEMO | Hpx | Hemopexin | -0.55 ** | -0.25 * | 0.31 ** |
| Q9CR20 | IR3IP | Ier3ip1 | Immediate early response 3-interacting protein 1 | -0.17 ** | 0.09 * | 0.26 *** |
| Q9ET54 | PALLD | Palld | Palladin | -0.44 ** | -0.18 * | 0.25 *** |
| P12246 | SAMP | Apcs | Serum amyloid P-component | -0.73 ** | -0.48 ** | 0.25 ** |
| Q9ERI5 | JMJD6 | Jmjd6 | Bifunctional arginine demethylase and lysyl-hydroxylase JMJD6 | 0.66 ** | 0.32 * | -0.34 * |
| P23950 | ZFP36L1 | Zfp36l1 | mRNA decay activator protein ZFP36L1 | 0.99 *** | 0.39 ** | -0.61 *** |
| Q9CZ62 | CEP97 | Cep97 | Centrosomal protein of 97 kDa | 1.10 ** | 0.19 * | -0.91 ** |
| P09926 | SURF2 | Surf2 | Surfeit locus protein 2 | 1.50 ** | 0.40 * | -1.10 ** |

**p*< 0.05, ***p* < 0.01, ****p*< 0.001.
