# Supplementary material for: A TMT-based shotgun proteomics uncovers overexpression of thrombospondin 1 as a contributor in pyrrolizidine alkaloid-induced hepatic sinusoidal obstruction syndrome
Source: Arch Toxicol. 2022 Mar 31;96(7):2003–19. doi: 10.1007/s00204-022-03281-7 (PMC9151551; doi:10.1007/s00204-022-03281-7)
Supplement: Supplementary file 2 — Supplementary file2 (PDF 755 KB) [file 204_2022_3281_MOESM2_ESM.pdf]

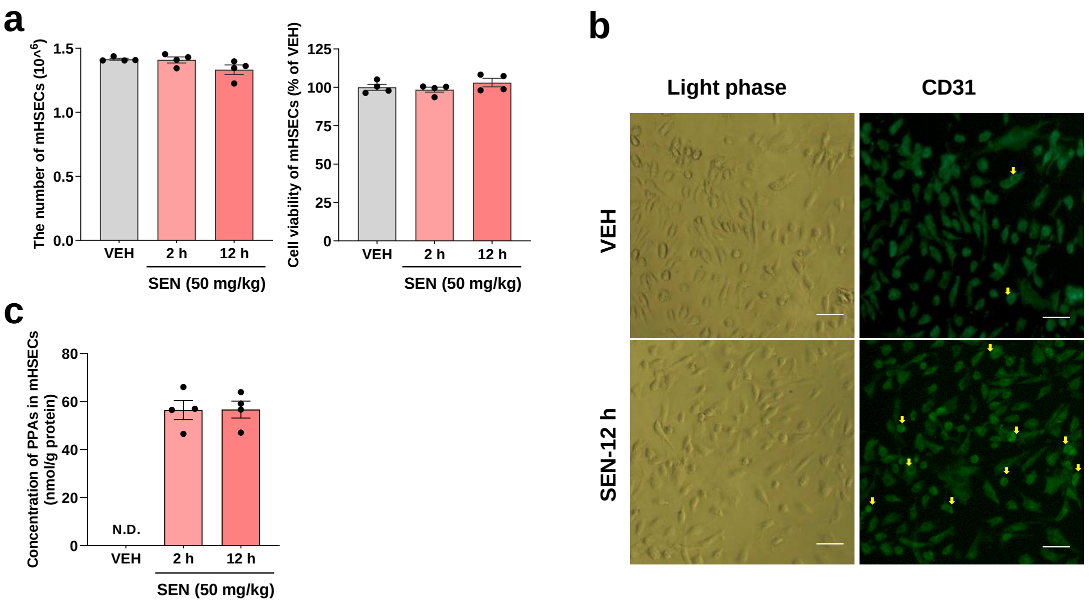

SI Fig. 1. Primary culture of mouse HSECs (mHSECs). Mice were orally treated with blank solvent (VEH) or senecionine (SEN, 50 mg/kg body weight) for 2 and 12 h, respectively ($n = 4$). The mHSECs were isolated by collagenase perfusion, metrizamide gradient centrifugation, and elutriation in accordance with the previous reported methods. **a** The yield of and viability of HSECs in mice upon VEH or SEN treatment. Values are expressed as the mean ± SEM ($n = 4$). **b** The identification of mHSECs. Scale bar: 20 μm. An aliquot of the purified mHSECs was seeded in 35 mm plates and immunologically stained with anti-endothelial cell antibody (CD31) in green. After attachment, the mHSECs in the dish showed a typical cobblestone, sheet-like appearance, and purity ~95%. Yellow arrow shows the surface CD31-positive cells. **c** Contents of PPAs in mHSECs isolated from mice upon VEH or SEN treatment. Values are expressed as the mean ± SEM ($n = 4$).

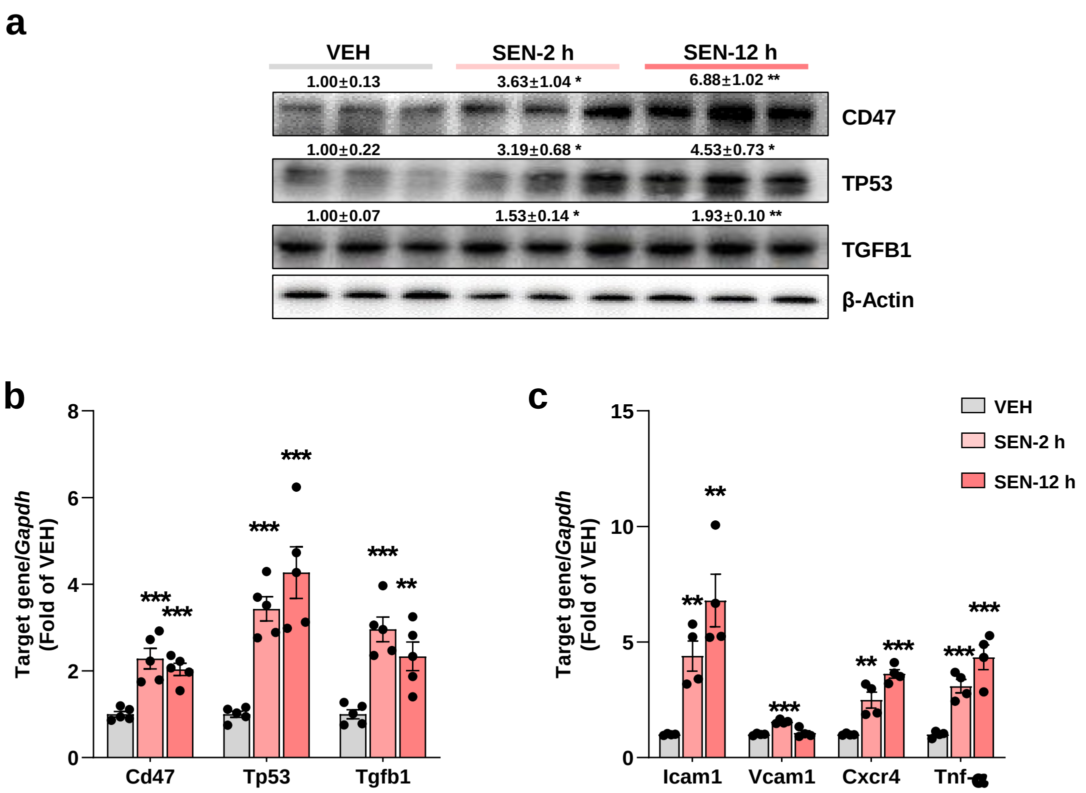

SI Fig. 2. Expression of selected genes and proteins in mHSECs isolated from mice. **a** The protein expression levels of CD47, TP53, and TGFB1 in mHSECs (*n*=3). **b** The mRNA expression levels of *Mmp9*, *Cd47*, *Tp53*, and *Tgfb1* in mHSECs (*n*=4). **c** The mRNA expression levels of pro-inflammatory cytokines in mHSECs (*n*=4). Values are expressed as the mean ± SEM. *$p < 0.05$, **$p < 0.01$, ***$p < 0.001$ vs. VEH group. *Icam1*, intercellular cell adhesion molecule 1; *Vcam1*, vascular cell adhesion molecule 1; *Tnf-α*, tumor necrosis factor; *Cxcr4*, chemokine receptor 4.

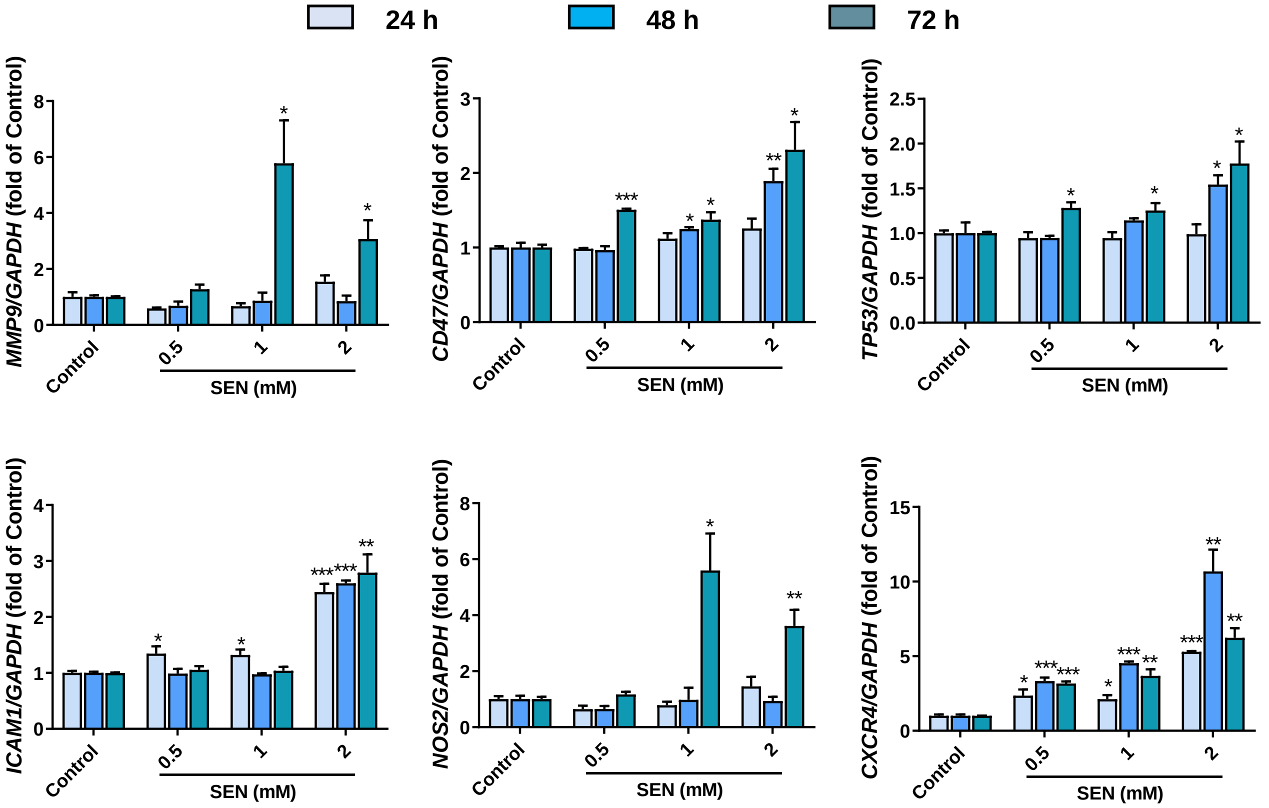

SI Fig. 3. Expressions of selected genes in human HSECs (hHSECs). The hHSECs were treated with blank solvent (control) or SEN (0.5, 1, and 2 mM) for 24, 48, and 72 h. Values are expressed as the mean ± SEM ($n = 3$). *$p < 0.05$, **$p < 0.01$, ***$p < 0.001$ vs. VEH group. *ICAM1*, intercellular cell adhesion molecule 1; *CXCR4*, chemokine receptor 4; *NOS2*, nitric oxide synthase 2. Three independent experiments were performed.

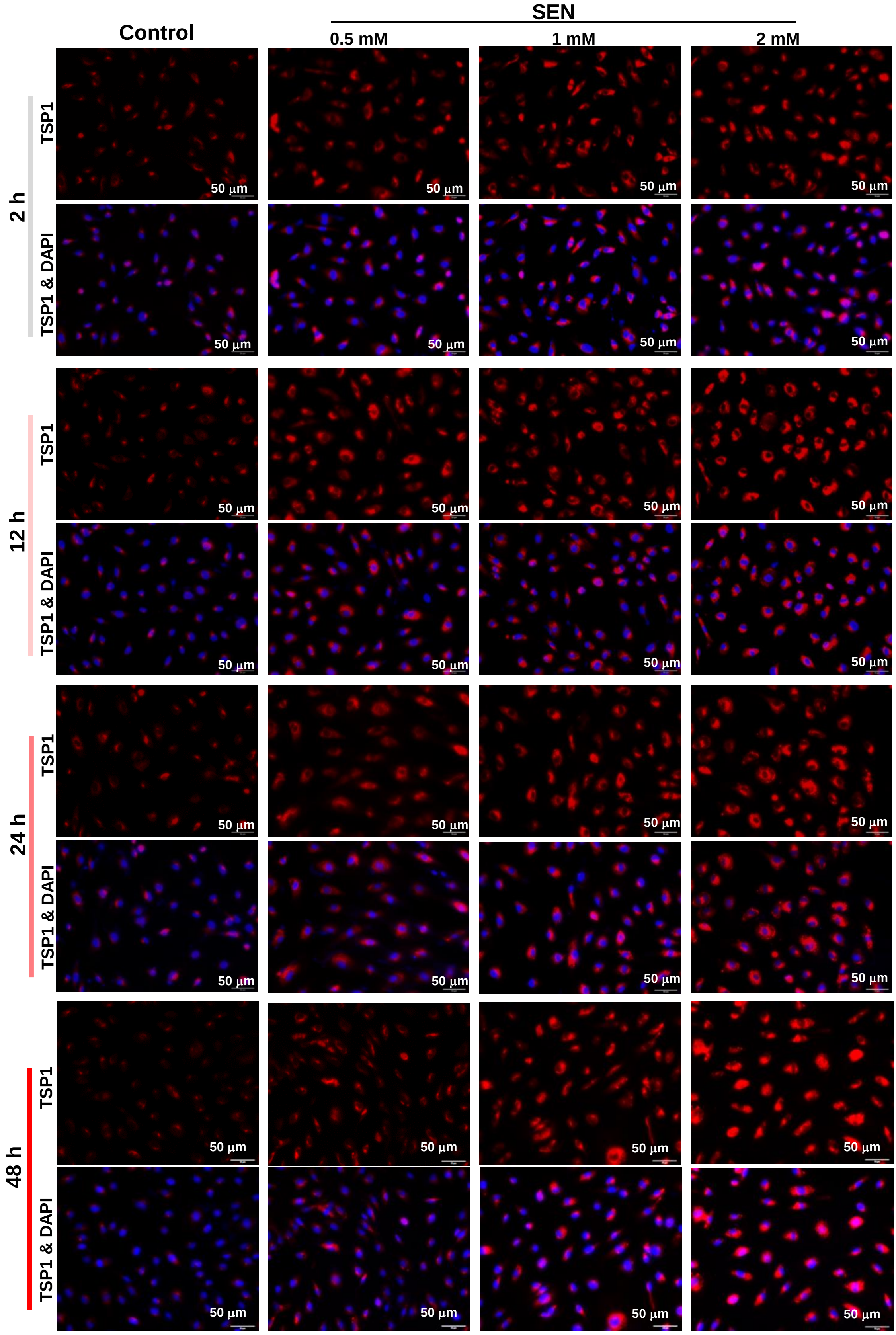

SI Fig. 4. Representative immunocytochemical staining images of TSP1 in hHSECs. Scale bar: 50 μm. The hHSECs were treated with blank solvent (control) or SEN (0.5, 1, and 2 mM) for 2, 12, 24, and 48 h. The cells were then immunologically stained with TSP1 in red and DAPI in blue. Three independent experiments were performed.

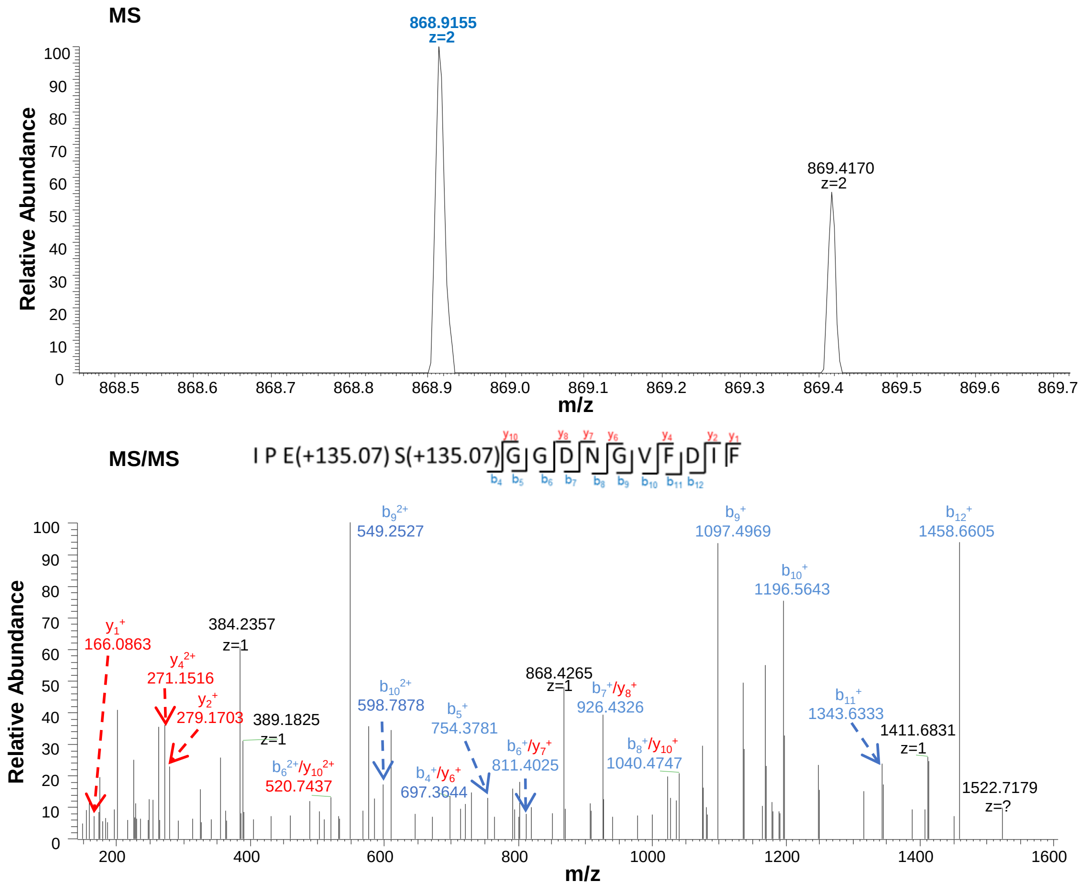

SI Fig. 5. MS spectrum showing the potential modification sites of DHP (135.0684 Da) in TSP1 in mouse livers after senecionine treatment (50 mg/kg bodyweight) for 12 h. The upper panel shows the MS spectrum of mouse TSP1. The lower panel shows a higher energy collision-induced dissociation (HCD) MS/MS spectrum recorded on the $[M+2H]^{2+}$ ion at m/z 868.9155 of the mouse TSP1 peptide IPESGGDNGVFDIF harboring two DHP site. Predicted b- and y-type ions (not including all) are listed below and above the peptide sequence, respectively. Matched ions are labeled in the spectrum and indicate that TSP1 is modified on E23 and S24.
